# Engineering Macrophage via Biomaterial-Mediated Mitochondrial Regulation: Mechanisms and Strategies

**DOI:** 10.34133/research.0883

**Published:** 2025-11-18

**Authors:** Jieliu Li, Minyu He, Shucheng Wan, Si Wang, Nanxin Liu, Liangjing Xin, Tao Chen

**Affiliations:** Stomatological Hospital of Chongqing Medical University, Chongqing Key Laboratory of Oral Diseases and Biomedical Sciences, Chongqing Municipal Key Laboratory of Oral Biomedical Engineering of Higher Education, Chongqing Medical University, Chongqing 401147, P. R. China.

## Abstract

Precisely targeting mitochondria to regulate macrophage fate has emerged as a critical therapeutic paradigm for managing inflammation-related pathologies. Mitochondria, while known for producing ATP, have been increasingly recognized for their critical involvement in immune cell differentiation and activation. As key innate immune effectors, macrophages dynamically adapt to microenvironmental cues through metabolic reprogramming and phenotype polarization, processes fundamentally controlled by mitochondrial homeostasis. Organelle-specific therapeutic advances now position mitochondria-targeted strategies as precision interventions with spatiotemporal advantages over conventional approaches. Crucially, these rationally designed systems demonstrate remarkable potential not only to direct macrophage differentiation toward anti-inflammatory phenotypes but also to reprogram the immune microenvironment concurrently, thereby achieving a breakthrough in precision medicine for inflammatory disorders. This review analyzes mitochondrial homeostasis mechanisms in pathophysiology, establishing design principles for targeted therapies. We classify emerging mitochondrial modulation approaches into indirect regulation and direct targeting, evaluating their impacts on macrophage plasticity and therapeutic efficacy. Critical translational challenges are examined, including single-cell-centric regulation, the complexity of mitochondrial interactions in macrophages, and the inefficiency of traditional trial-and-error strategies. The proposed artificial intelligence (AI)-driven methods such as deep learning-based material design, metabolic network modeling, and advanced small-molecule synthesis can accelerate the development of targeted mitochondrial therapies and enhance clinical feasibility. This synthesis aims to accelerate the development of mitochondrially engineered immunotherapies through rational design principles and standardized evaluation protocols.

## Introduction

As master regulators of immune homeostasis, macrophages exert critical regulatory functions spanning innate defense mechanisms to tissue repair processes through their remarkable phenotypic plasticity [1–3]. They are active participants in all stages of the inflammatory process [4]. The classical M1/M2 polarization paradigm, while oversimplified, remains instrumental in understanding these phagocytes’ dichotomous roles in disease progression—from perpetuating inflammatory cascades to orchestrating resolution pathways [5]. However, dysregulation of macrophage fate frequently arises in diseases such as pneumonia, cardiovascular diseases, and inflammatory bowel disease, compromising therapeutic outcomes [6–8]. Consequently, precise modulation of macrophages in disease contexts presents an urgent clinical imperative.

Emerging evidence establishes mitochondrial homeostasis as the central rheostat governing macrophage fate determination, where organellar integrity directly dictates inflammatory commitment versus reparative transformation [9–11]. Mitochondria, as integral components of the respiratory chain, generate substantial adenosine triphosphate (ATP) through oxidative phosphorylation (OXPHOS), providing a continuous energy supply for cellular functions that is closely linked to macrophage polarization [11–13]. Structurally, mitochondria are double-membrane-bound organelles, where the outer mitochondrial membrane (OMM) and inner mitochondrial membrane (IMM) compartmentalize the organelle into the matrix and intermembrane space [14]. These membranes regulate ion and molecular trafficking via selective permeability and active transport mechanisms [15]. Beyond energy production, mitochondria are pivotal in diverse processes, including quality control, mitochondrial reactive oxygen species (mtROS) generation, ion homeostasis, and mitochondria–endoplasmic reticulum (ER) interactions [16]. Notably, mitochondria are semi-autonomous organelles harboring their own genome (mtDNA). While mtDNA primarily encodes components of the mitochondrial electron transport chain (ETC), it also plays a critical role in modulating macrophage immune responses [17]. For instance, mtDNA released into the cytosol under stress acts as a damage-associated molecular pattern (DAMP), activating inflammatory pathways such as cGAS-STING and NLRP3 inflammasomes [18]. Targeting mitochondrial pathways holds therapeutic potential for regulating macrophage fate, including polarization, functional plasticity, and survival.

The deepening understanding of mitochondria as signaling integrators in macrophage polarization has propelled mitochondrial medicine to the forefront of immunotherapy innovation [19,20]. While mitochondria serve as the central signaling hub coordinating macrophage metabolism, epigenetics, and inflammatory responses, their double-membrane architecture presents unique pharmaceutical challenges [21]. This makes precise mitochondrial targeting challenging. Given the critical role of mitochondria in regulating macrophages, reviewing strategies for targeted mitochondrial regulation is essential.

**Fig. 1.**
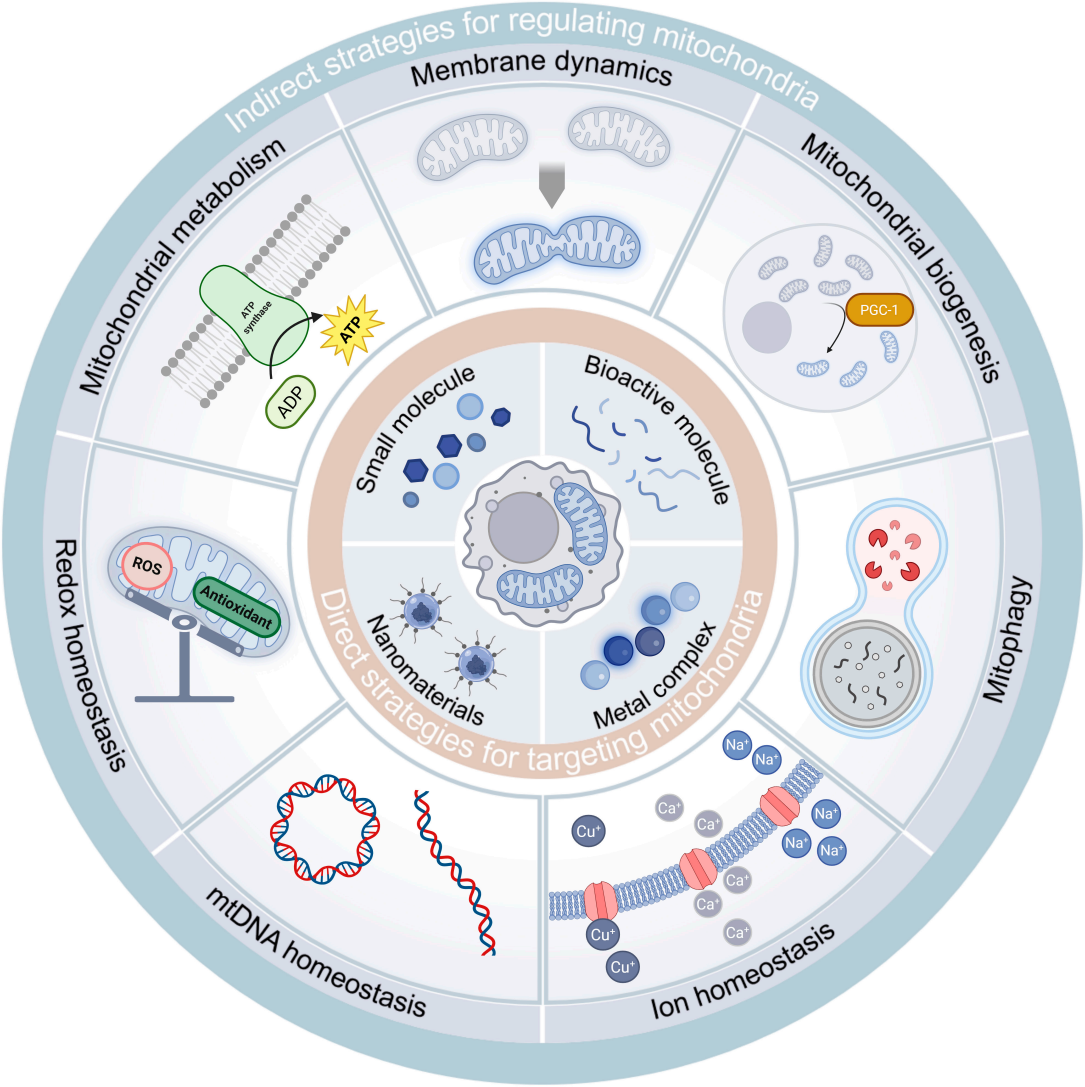
Indirect and direct strategies for targeting mitochondria to modulate macrophage fate. Four major mitochondrial-targeting materials of direct strategies endow targeting strategies with the ability to precisely regulate mitochondrial function. Indirect regulatory strategies influence macrophage polarization by restoring the balance of mitochondrial quality control, metabolism, mtROS, mitochondrial ion, and mtDNA (created with BioRender.com).

This comprehensive review systematically examines the intricate relationship between mitochondrial homeostasis and macrophage immunobiology. We first elucidate the mechanistic underpinnings of mitochondrial homeostasis in governing macrophage functional plasticity and phenotypic determination. Subsequently, we present a critical taxonomy of mitochondrial regulatory strategies, categorizing current interventions according to their homeostatic modulatory mechanisms while providing an objective appraisal of their therapeutic merits and technical constraints. Building on this foundation, we critically evaluate emerging pharmacological and bioengineering interventions capable of precise mitochondrial targeting, with particular emphasis on their immune-metabolic reprogramming capabilities. Our analysis culminates in a rigorous assessment of current translational challenges and emerging opportunities in macrophage-centric mitochondrial therapeutics. We posit that mitochondrial-targeted immunomodulation represents a paradigm-shifting approach in cellular medicine, offering unprecedented opportunities for developing pathophysiology-driven treatment modalities. This strategic intervention axis not only expands our therapeutic arsenal for complex disorders but also provides a robust conceptual framework for advancing precision medicine paradigms, ultimately paving the way for transformative developments in targeted immunotherapy (Fig. 1).

## Mitochondria: The Regulators of Macrophage Fate

Mitochondria, ubiquitous double-membraned organelles found in nearly all eukaryotic cells, are hypothesized to have originated from an ancient endosymbiotic fusion between bacteria and proto-eukaryotic cells [22]. Structurally, its dual membrane system—comprising an OMM and a highly folded IMM—compartmentalizes distinct functional domains. The OMM contains transporter proteins such as voltage-dependent anion channel/porin (VDAC) as well as translocase of the outer membrane channel, which facilitate the vital exchange of ions and proteins essential for mitochondrial homeostasis [23]. Separated by the intermembrane space, the IMM embeds a sophisticated protein machinery, such as the ETC complexes and the mitochondrial calcium uniporter (MCU), which collectively underpin its multifaceted roles [24,25]. Functionally, our understanding of mitochondria has evolved from their traditional role as cellular “powerhouses” to dynamic hubs coordinating biosynthesis, signaling, and metabolic regulation [26]. This paradigm shift is driving the transition from mitochondrial medicine to mitochondria-centric precision medicine, emphasizing tailored interventions based on mitochondrial dynamics.

### Mitochondrial quality control

Mitochondria operate within a multilayered quality control system to preserve cellular homeostasis [27]. To sustain functional mitochondrial networks and ensure uninterrupted aerobic energy production, cells must coordinate 3 tightly regulated processes: (a) recognition of irreversibly damaged mitochondria, (b) their targeted elimination via selective mitophagy, and (c) replenishment through mitochondrial biogenesis [28]. Disruption of this quality control cycle not only impairs energy metabolism but also triggers cascading cellular pathologies, including calcium (Ca^2+^) dyshomeostasis, dysregulated heme biosynthesis, oxidative stress from pathological mtROS overproduction, and activation of intrinsic apoptotic pathways.

#### Mitochondrial membrane dynamics

Mitochondria exhibit evolutionarily conserved structural plasticity, characterized by dynamic changes in organellar morphology, ranging from elongated networks to fragmented forms, and ultrastructural features such as cristae density and membrane curvature. This adaptive remodeling, influenced by cell cycle phase, metabolic demands, and stress signals, forms the biological basis of mitochondrial dynamics, a homeostatic system regulated by 2 opposing processes: network integration via fusion and quality control through fission [29]. The fusion machinery employs guanosine triphosphatase (GTPase)-mediated membrane remodeling through stage-specific effectors, mitofusins (MFN1/2), executing OMM fusion [30,31], followed by optic atrophy 1 (OPA1)-mediated IMM fusion [32,33]. This process enables content homogenization across the mitochondrial population, facilitating functional complementation between organelles. Conversely, fission events directed by dynamin-related protein 1 (DRP1) involve its oligomerization at ER–mitochondria contact sites, culminating in membrane constriction and daughter organelle formation, thereby permitting selective segregation of damaged components [34,35]. Cristae architecture, the IMM’s functionally specialized subdomain, undergoes activity-dependent topological reorganization mediated by OPA1 isoforms and MICOS complex proteins [36–38]. These structural adaptations regulate respiratory efficiency through 3 key mechanisms: (a) optimizing supercomplex assembly in tightly folded cristae [39], (b) sequestering cytochrome c within cristae junctions to prevent premature apoptosis [40], and (c) aligning ATP synthase dimers along cristae ridges for vectorial proton transport [41]. Notably, mitochondrial ultrastructural plasticity directly dictates macrophage polarization states [42]. Pro-inflammatory M1 macrophages exhibit fission-dominant fragmented networks with dilated cristae, correlating with glycolytic dependency. In contrast, reparative M2 counterparts maintain fusion-elongated mitochondria containing densely packed cristae that maximize OXPHOS efficiency. This bidirectional regulation establishes mitochondrial dynamics as a metabolic rheostat controlling macrophage functional commitment.

#### Mitophagy

The autophagic system orchestrates selective clearance of dysfunctional mitochondria through lysosomal degradation, a conserved quality control mechanism termed mitophagy [43]. This process dynamically regulates mitochondrial homeostasis by balancing mitochondrial biogenesis with lysosomal degradation, thereby maintaining functional organelle populations [44,45]. Mitophagy execution is primarily mediated by OMM receptors including NIX/BNIP3L, BNIP3, and FUNDC1, which possess a conserved light chain 3 (LC3)-interacting region (LIR) motif that directly binds microtubule-associated protein LC3 on autophagosomal membranes [46–48]. In parallel, the PINK1–Parkin pathway serves as another critical regulatory axis for mitophagy initiation [49,50]. Mitochondrial depolarization triggers stabilization of PINK1 on the outer membrane, which phosphorylates ubiquitin chains and recruits cytosolic Parkin E3 ligase [51,52]. This cascade culminates in extensive ubiquitination of mitochondrial substrates, facilitating ubiquitin-dependent recruitment of autophagic receptors that mediate LC3 binding and subsequent autophagosome encapsulation [53].

Mitophagy serves as a metabolic checkpoint governing macrophage phenotypic commitment through mitochondrial quality control. During M1 polarization, activated mitophagy removes damaged mitochondria to support pro-inflammatory glycolysis [54]. This clearance mechanism ensures optimal expression of glycolytic enzymes and sustains cytokine production, with deficient macrophages showing impaired inflammation [44,55]. Paradoxically, hyperglycemic conditions disrupt mitochondrial clearance machinery while paradoxically amplifying M1 activation, suggesting glucose concentration-dependent modulation of mitophagy’s immune-metabolic regulation [56]. The bidirectional crosstalk between mitophagic flux and metabolic states ultimately dictates macrophage fate determination: Efficient mitochondrial turnover favors classical M2 reparative differentiation, whereas defective mitophagy promotes alternative M1 pro-inflammatory phenotypes through accumulated mitochondrial DAMPs [44,57]. This regulatory axis positions mitophagy as a rheostat balancing immune activation resolution and metabolic homeostasis in macrophage biology.

#### Mitochondrial biogenesis

Mitochondrial biogenesis establishes cellular energetic capacity through coordinated expansion of the organelle network, serving as the quality control counterbalance to mitophagic elimination [27]. Since mitochondria cannot be generated de novo, their biosynthesis relies on the dynamic integration with existing mitochondrial structures. This process involves membrane remodeling and content replication, coordinated by the collaboration of both genomes: Nuclear DNA encodes the structural components, while mtDNA encodes the OXPHOS subunits [58]. The process is activated by metabolic sensors including adenosine monophosphate–activated protein kinase (AMPK), sirtuin 1 (SIRT1), and mechanistic target of rapamycin (mTOR), which integrate intracellular energy status {ATP/ADP (adenosine diphosphate) ratio, NAD^+^ [nicotinamide adenine dinucleotide (oxidized form)]/NADH (reduced form of NAD^+^) levels} with extracellular cues like nutrient availability and oxidative stress [59]. This multilayered regulatory architecture converges on master transcriptional coactivators, principally the peroxisome proliferator-activated receptor γ coactivator (PGC-1) family [60]. PGC-1α operates as the nodal orchestrator by (a) partnering with nuclear respiratory factors (Nrf1/2) to activate mitochondrial transcription factor A (TFAM)-dependent mtDNA replication [61], (b) coordinating with estrogen-related receptors (ERRα/γ) to up-regulate OXPHOS complexes [62,63], and (c) synergizing with antioxidant transcription factors (FOXO3, Nrf2) to sustain mitochondrial redox homeostasis [64]. Through this tripartite regulatory axis, PGC-1α simultaneously activates metabolic genes favoring β-oxidation while suppressing pro-inflammatory signaling. This dual functionality calibrates mitochondrial mass to both energetic demands and inflammatory tone [65].

Mitochondrial biogenesis functions as an immune-metabolic rheostat in macrophage phenotypic polarization, dynamically integrating inflammatory signaling with organelle replenishment. Mechanistically, macrophage-derived nitric oxide (NO) and compartmentalized ROS activate AMPK/SIRT1-mediated PGC-1α signaling and Nrf2-dependent heme oxygenase-1 (HO-1) induction [66]. This dual redox-sensitive pathway couples de novo mitochondrial biosynthesis with glutathione regeneration systems, thereby resolving inflammation through synchronized antioxidant defense activation and damaged organelle replacement. The biosynthetic–metabolic coordination proves critical during inflammatory challenges through 3 interlocking mechanisms: New mitochondrial networks sustain NADPH (reduced form of nicotinamide adenine dinucleotide phosphate) production to fine-tune phagocytic burst dynamics while simultaneously resolving oxidative stress [67]; concurrently, biogenesis-coupled HO-1 activity converts pro-inflammatory heme into anti-inflammatory biliverdin, thereby neutralizing cytotoxic iron accumulation [68]; this redox-regulatory axis synergizes with PGC-1α/Nrf2-mediated cardiolipin remodeling in mitochondrial membranes, which directly suppresses NLRP3 inflammasome oligomerization by stabilizing cristae architecture and preventing oxidized lipid release [69]. Additionally, the promotion of mitochondrial biogenesis enhances mitochondrial energy production and ATP synthesis, which is critical for sustaining immune cell functions and controlling excessive inflammatory responses [70]. By supporting mitochondrial renewal, these processes help to optimize the balance between cellular metabolism and inflammatory resolution. Conversely, impaired biogenesis disrupts mitochondrial membrane potential oscillations, trapping macrophages in a pro-inflammatory loop marked by mtDNA leakage and STING-dependent interferon responses [71]. This self-reinforcing cycle establishes mitochondrial biogenesis capacity as a determinant of macrophage fate specification. Sufficient organelle replenishment promotes resolution-phase transition to pro-repair M2-like states, driven by metabolic shifts such as increased OXPHOS and fatty acid oxidation [69]. In contrast, biogenesis failure perpetuates M1 hyperactivation, characterized by glycolytic dominance and inflammatory cytokine production, leading to sustained tissue damage [69]. Through these mechanisms, macrophage mitochondrial biosynthesis emerges as a regulator balancing pro-resolving inflammation with tissue-protective antioxidant responses.

### Mitochondrial metabolism

As the cell’s bioenergetic nexus, mitochondria execute coordinated substrate oxidation through the tricarboxylic acid (TCA) cycle and ETC, coupling redox reactions with proton gradient-driven ATP synthesis via OXPHOS. This sophisticated energy transduction system generates over 30 ATP molecules per glucose molecule, its efficiency increasing 15-fold compared to cytosolic aerobic glycolysis, which yields only 2 ATP. Beyond mere energy factories, mitochondrial metabolism produces critical regulatory intermediates: TCA cycle-derived citrate regulates lipid synthesis, while succinate and acetyl-CoA (coenzyme A) serve as epigenetic modifiers influencing macrophage polarization [72,73]. The system’s supremacy stems from dynamic metabolite shuttling—Glycolytic pyruvate enters mitochondria as acetyl-CoA for complete oxidation, whereas cytoplasmic NADH transfers reducing equivalents through malate-aspartate and glycerol phosphate shuttles [74]. When mitochondrial respiration is compromised, cells resort to inefficient glycolytic ATP production, accumulating lactate as a result of anaerobic fermentation, and depleting NAD^+^ pools through the conversion of pyruvate to lactate [75]. This metabolic reprogramming triggers compensatory mitophagy and the accumulation of α-ketoglutarate (α-KG) and fumarate, which contribute to ROS production by disrupting mitochondrial electron transport and modulating redox-sensitive pathways, thereby amplifying inflammatory signaling [76]. Crucially, mitochondrial OXPHOS maintains cellular ATP turnover rates matching physiological demands through allosteric regulation of rate-limiting dehydrogenases by NADH/ATP ratios [77]. Given ATP’s limited buffering capacity, mitochondrial respiratory control via uncoupling proteins (UCPs) and adenine nucleotide translocase becomes indispensable for preventing energy crises [78]. These features establish mitochondrial metabolic flux not only as an energy source but also as the primary rheostat governing cellular redox balance, biosynthetic precursor supply, and inflammatory signaling cascades.

Mitochondrial metabolic reprogramming dictates macrophage functional polarization through dynamic substrate utilization and effector metabolite production [79]. M1 macrophages undergo aerobic glycolysis dominance characterized by hypoxia-inducible factor-1α (HIF-1α)-mediated pyruvate dehydrogenase (PDH) kinase up-regulation, which shunts pyruvate toward lactate accumulation while suppressing TCA cycle flux [80]. This Warburg-like metabolic shift generates itaconate via IRG1-mediated cis-aconitate decarboxylation, a pleiotropic immunometabolite that both inhibits succinate dehydrogenase (SDH) to stabilize HIF-1α and alkylates KEAP1 to activate Nrf2 antioxidant responses [81,82]. Concomitant mitochondrial membrane potential dissipation reduces OXPHOS efficiency, creating a self-amplifying loop through accumulated TCA cycle intermediates—succinate-driven prolyl hydroxylase (PHD) inhibition further stabilizes HIF-1α, while citrate export to cytoplasm fuels NO and ROS synthesis via inducible nitric oxide synthase (iNOS) and NADPH oxidase 2 (NOX2) [83]. Conversely, M2 polarization engages peroxisome proliferator–activated receptor γ (PPARγ)-dependent fatty acid uptake and CPT1a-mediated β-oxidation, feeding acetyl-CoA into a reconfigured TCA cycle that sustains OXPHOS-driven ATP production [84]. This metabolic architecture generates α-KG to demethylate histone H3K27me3 through Jumonji domain-containing dioxygenases, epigenetically reinforcing interleukin-10 (IL-10) transcription [85]. Notably, mitochondrial cristae stabilization in M2 macrophages permits efficient ETC coupling. This process generates NAD^+^ to fuel SIRT3-dependent deacetylation of IDH2. This redox optimization mechanism reciprocally enhances fatty acid oxidation [86]. Through these dichotomous metabolic circuits, mitochondrial metabolites operate as both energy currencies and epigenetic modifiers, establishing organellar metabolism as the central processing unit of macrophage phenotypic commitment.

### Mitochondrial ROS

Mitochondrially generated reactive oxygen species (ROS) encompassing superoxide (O_2_•^−^), hydrogen peroxide (H₂O₂), and hydroxyl radicals (•OH) serve dual physiological roles. They function as essential redox signaling mediators while also acting as potential causes of macromolecular damage [87]. At physiological levels, these reactive molecules participate in homeostatic signaling [88], yet their dysregulated propagation triggers oxidative cascades that peroxidize lipids, carbonylate proteins, and fragment mtDNA [89]. In activated macrophages, mtROS production arises primarily from electron leakage from the ETC [90]. Counterbalancing this generation is a tripartite antioxidant defense system: Manganese superoxide dismutase (MnSOD) initiates detoxification by dismutating O_2_•^−^ to H₂O₂ [91], which glutathione peroxidase 4 (GPX4) then reduces to water using glutathione (GSH) as an electron donor [92], while peroxiredoxin III (PrxIII) concurrently neutralizes peroxynitrite through cyclic thiol oxidation–reduction reactions [93]. The dynamic equilibrium of this redox landscape is governed by the mitochondrial transmembrane potential (Δψm), an electrochemical gradient established through directional proton pumping during ETC activity. Δψm regulates redox homeostasis through biphasic control of mtROS generation. Physiological membrane potentials (−140 mV to −180 mV) permit complex I (CI)-derived transient O_2_•^−^ bursts that activate cytoprotective pathways [94], whereas hyperpolarization (below −200 mV) induces electron congestion at complex III (CIII) quinone sites, remarkably increasing ROS production [95]. This voltage-sensitive ROS modulation is fine-tuned by UCPs, which mitigate excessive Δψm through controlled proton reflux, thereby establishing a self-correcting feedback loop [96]. Through this spatially organized bioenergetic–redox crosstalk, mitochondria calibrate ROS fluxes to synchronize macrophage inflammatory responses with oxidative damage containment, exemplifying their role as metabolic sentinels in immune regulation.

mtROS function as pleiotropic signaling hubs in macrophage immunometabolism, directly coupling OXPHOS dynamics to inflammatory output through multi-tiered regulatory circuits. Mechanistically, mtROS activate the mitogen-activated protein kinase (MAPK)/nuclear factor κB (NF-κB) axis via oxidation of critical cysteine residues in IκB kinase γ (IKKγ) and ASK1 phosphatase thioredoxin-1, driving transcriptional up-regulation of tumor necrosis factor-α (TNF-α), IL-6, and cyclooxygenase-2 (COX-2) [97]. Concurrently, mtROS stabilize HIF-1α through 2 synergistic mechanisms: inhibition of PHDs via ascorbate depletion and oxidation of Fe^2+^ in PHD catalytic centers, collectively promoting glycolytic enzyme transcription while suppressing PDH-mediated pyruvate entry into the TCA cycle [98]. This metabolic rewiring sustains LPS-induced IL-1β maturation through mtROS–NLRP3 inflammasome crosstalk—oxidized mitochondrial cardiolipin recruits NLRP3 to mitochondrial membranes, while K^+^ efflux caused by ETC-derived ROS potentiates apoptosis-associated speck-like protein containing a CARD (ASC) speck assembly [99,100]. In parallel, mtROS-induced oxidative stress facilitates STING activation by amplifying STING-dependent IFN signaling, thereby modulating macrophage inflammatory responses [101]. Furthermore, mtROS-driven activating transcription factor 4 (ATF4)–C/EBP homologous protein (CHOP) signaling activates the mitochondrial unfolded protein response (UPRmt), contributing to macrophage functional reprogramming and the regulation of inflammatory resolution [102]. Mechanistically, focal plasma membrane photodamage triggers DRP1-mediated mitochondrial fission at injury sites, with resultant ROS bursts facilitating membrane repair through lipid peroxidation signaling [103]. Collectively, these spatially constrained ROS microdomains exemplify how mitochondrial information processing systems orchestrate gene expression patterns and subcellular functions through compartmentalized redox signaling.

### Mitochondrial ion homeostasis

Mitochondrial ion flux operates as an electrochemical decoder in macrophage functional programming, translating extracellular immune signals into organellar metabolic responses through voltage-gated ionic circuits. The IMM potential establishes a biophysical platform for charged species sensing, with Ca^2+^ serving as the predominant second messenger [104]. The MCU mediates activity-dependent Ca^2+^ influx within milliseconds. This influx couples cytosolic Ca^2+^ oscillations to TCA cycle amplification by activating PDH phosphatase, which gates pyruvate entry into oxidative metabolism [105,106]. This Ca^2+^-metabolism nexus directly modulates macrophage inflammatory polarization. MCU deficiency blunts LPS-induced itaconate production in macrophages through attenuated α-KG dehydrogenase activity, thereby impairing NLRP3 inflammasome resolution [107,108]. The mitochondrial Na^+^/Ca^2+^ exchanger (NCLX) introduces spatiotemporal regulation by extruding matrix Ca^2+^ in exchange for cytosolic Na^+^, maintaining ionic homeostasis critical for mitochondrial permeability transition pore (mPTP) gating [109]. In macrophages, NCLX activity prevents Ca^2+^-induced cristae remodeling during phagocytosis, preserving ETC integrity for sustained ROS production [110]. Simultaneously, accumulated matrix Na^+^ activates NHE1 to mediate proton export, thereby promoting pH-dependent mtDNA release, which is a key event for STING pathway activation [111]. This dual-ion rheostat (Ca^2+^/Na^+^) coordinates metabolic flexibility with danger signaling [24]. Elevated Na^+^/Ca^2+^ ratios promote fatty acid oxidation-driven M2 polarization through SIRT3 deacetylation of IDH2. In contrast, Ca^2+^-dominant states favor glycolytic M1 commitment via CaMKII–HIF-1α signaling.

As multimodal ionic sentinels, mitochondria employ an evolutionarily conserved toolkit of transporters and redox-sensitive modifications to dynamically interpret extracellular ionic milieus. Beyond canonical Ca^2+^ sensing via MCU, mitochondria detect monovalent ions such as Na^+^ through NCLX [109] and Cl^−^ via putative SLC25A51 paralogs [112], while transition metals like Fe^2+^ are monitored via mitoferrin-dependent import coupled to frataxin-mediated storage [113], and Cu^2+^ through CTR1/ATP7B-regulated transport coordinated with chaperone-mediated sequestration [114]. Ferroptosis is a form of iron-dependent cell death closely linked to mitochondrial iron homeostasis. Dysregulation of iron import via mitoferrin and storage by frataxin leads to iron overload, causing lethal lipid peroxidation through ROS accumulation and GPX4 inactivation [115]. In macrophages, ferroptotic stress enhances pro-inflammatory signaling by inducing mtROS bursts that activate the NLRP3 inflammasome, leading to IL-1β and IL-18 secretion and M1 polarization [116]. Additionally, lipid peroxides released from damaged mitochondria act as DAMPs that activate neighboring macrophages through Toll-like receptor 4 (TLR4) and NF-κB pathways [117]. Building upon the mitochondrial-centric mechanisms of ferroptosis in macrophages, which hinge on iron-mediated lipid peroxidation and inflammasome activation, a parallel yet distinct pathway emerges in the context of copper dysregulation. Cuproptosis is a copper-dependent cell death pathway that is driven by mitochondrial copper overload, which results from dysregulated CTR1/ATP7B transport. This process induces proteotoxic stress by aggregating lipoylated TCA cycle enzymes and remodeling cristae via direct copper–sulfur interactions [118]. In macrophages, this copper overload disrupts mitochondrial energy metabolism by crippling ATP production and NAD^+^/NADH homeostasis, forcing a glycolytic shift that stabilizes HIF-1α and entrenches pro-inflammatory M1 polarization [119]. Copper-induced cristae fragmentation further releases mitochondrial DNA into the cytosol, activating the cGAS-STING axis, leading to hyperinflammatory macrophage states [120]. Even low-abundance ions such as Li^+^ may modulate IMM phospholipid packing through charge-shielding effects, although their dedicated transporters remain uncharacterized [121].

In summary, the precise regulation of key mitochondrial ions including Ca^2+^, Na^+^, Fe^2+^, and Cu^2+^ extends beyond energy production and redox homeostasis to directly modulate macrophage polarization and immune signaling pathways. This underscores the critical role of mitochondrial ion dynamics in shaping immune responses and highlights the organelle’s capacity to decode extracellular cues into precise cellular outcomes, ultimately determining macrophage function in health and disease.

### mtDNA

The mitochondrial genome operates as a dual-functional entity: a genetic repository encoding 13 OXPHOS subunits, 22 transfer RNAs (tRNAs), 2 ribosomal RNAs (rRNAs), and regulatory mitochondria-derived peptides, and a stress-responsive hub sensitive to extracellular perturbations such as mutagens and nucleotide pool imbalances [122]. This genomic vulnerability stems from 3 structural constraints: close exposure to ROS, lack of histone protection, and error-prone replication [123]. These factors allow mtDNA damage to directly disrupt mitochondrial homeostasis. Both germline and somatic mtDNA mutations impair OXPHOS complex assembly, propagating a cascade of bioenergetic failure: diminished ETC efficiency, collapse of Δψm, and systemic metabolic dysregulation [124]. As Δψm and OXPHOS regulate key mitochondrial functions including metabolite transport, redox balance, and ion gradient maintenance, their disruption leads to ATP depletion, oxidative stress, and immune reprogramming.

Beyond these primary defects, mtDNA lesions propagate systemic metabolic chaos. In skeletal muscle, for instance, focal mtDNA deletions not only cripple respiratory enzyme function but also provoke adaptive mitochondrial proliferation—a compensatory mechanism to offset energetic deficits [125]. Even nonpathogenic mtDNA polymorphisms exert pleiotropic effects, altering metabolite flux to generate phenotypic diversity that modulates aging and disease trajectories [126]. The inherent genomic fragility of mtDNA, caused by limited repair capacity and replication errors, necessitates robust surveillance systems to detect genotoxic stress as a primary defense mechanism.

In macrophages, mtDNA homeostatic failure directly dictates immune-metabolic reprogramming. Large-scale deletions impair CI assembly, triggering glycolytic dependency. This metabolic shift consequently enhances IL-1β secretion [127]. Concurrently, cytosolic mtDNA escape subverts mitochondrial quality control, activating cGAS-STING via DAMPs to drive IFN production and entrench M1 polarization [128]. Subtle mtDNA variants further distort NAD^+^/NADH ratios, compromising SIRT3-dependent deacetylation of IDH2 and ACLY, thereby changing inflammatory signaling [129].

## Indirect Strategies for Regulation of Mitochondria

### Membrane dynamics control: Shaping morphology

As the principal mechanochemical effector of mitochondrial fission, Drp1 orchestrates organelle division through membrane-anchored adaptor protein complexes, assembling constrictive superstructures that execute precise mitochondrial separation [130]. This mechano-enzymatic activity establishes Drp1 as a central regulator of mitochondrial dynamic homeostasis, positioning it as a promising therapeutic target for correcting organellar dysregulation in disease states.

The small-molecule Mdivi-1, a cell-permeable quinazolinone, has been identified as an inhibitor of Drp1 activity in both yeast and mammalian cells. By blocking Drp1 oligomerization, Mdivi-1 interferes with the GTPase activity required for mitochondrial division [131]. Recent work by Su et al. [132] demonstrated that Mdivi-1 reduces mitochondrial fragmentation and alleviates mitochondrial dysfunction by preventing Drp1 phosphorylation at Ser^616^ and its translocation to mitochondria. This reduction in mtROS production suppresses NLRP3 inflammasome activation and shifts macrophage polarization away from the pro-inflammatory M1 phenotype, offering potential therapeutic benefits in the treatment of atherosclerosis. Complementing this pharmacological strategy, baicalein, a bioactive flavonoid from *Scutellaria baicalensis* roots, achieves convergent regulation of mitochondrial dynamics via divergent molecular pathways [133]. Unlike Mdivi-1’s posttranslational modulation, baicalein down-regulates Drp1 expression at transcriptional and translational levels while retaining potent antioxidative and anti-inflammatory properties. Parallel investigations into traditional pharmacopeia reveal broader therapeutic potential, exemplified by *Thymus quinquecostatus* CELAK (TQC) extracts restoring LPS-disrupted mitochondrial morphology networks and associated gene expression, suggesting the evolutionary conservation of phytochemicals in regulating organellar homeostasis [134].

**Fig. 2.**
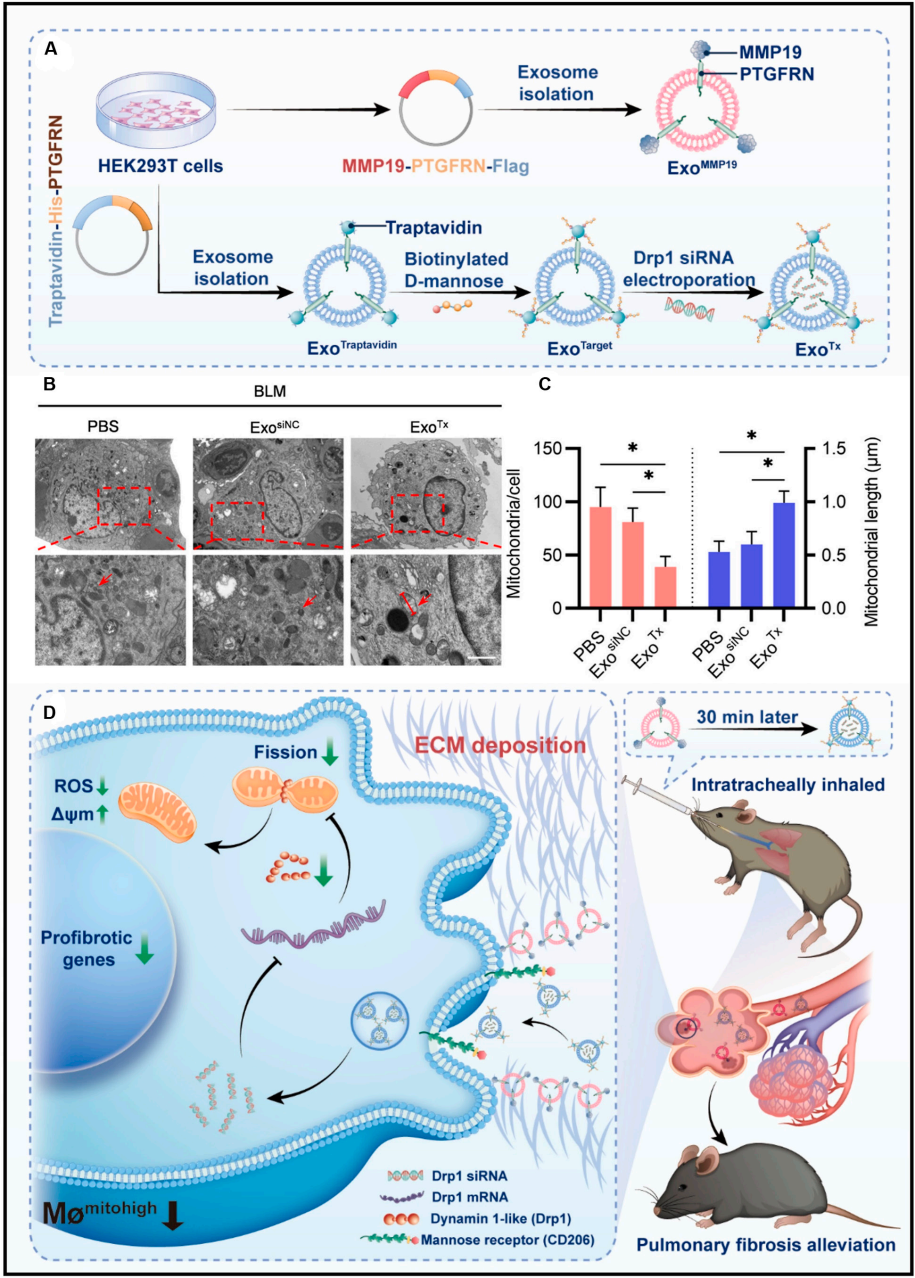
(A) Synthesis diagram of Exo^Tx^. (B) Representative transmission electron microscopy (TEM) images of mitochondrial morphology in lung macrophages treated with phosphate-buffered saline (PBS)/Exo^siNC^/Exo^Tx^. (C) Calculated mitochondria per cell and mitochondrial length. (D) Exo^Tx^ carrying siDrp1 inhibited mitochondrial fission and effectively alleviated pulmonary fibrosis [135]. Copyright 2024, KeAi.

Advancements in organelle-specific therapeutics have catalyzed innovative approaches for Drp1 modulation in macrophages. Zhang et al. [135] constructed a multi-functional drug delivery system (DDS) using engineered exosomes targeting macrophages for pulmonary fibrosis. A general-targeted exosomes were designed by fusing traptavidin with exosomal protein PTGFRN, which has been utilized as a surface display scaffold for structurally and biologically diverse proteins. They constructed Exo^Target^ by first generating Exo^Traptavidin^ through expression of traptavidin-His-PTGFRN in human embryonic kidney 293T (HEK293T) cells and isolating the resulting exosomes and then conjugating biotinylated d-mannose to the Exo^Traptavidin^ surface to enable specific targeting of CD206-positive macrophages (Fig. 2A). siDrp1 was loaded into Exo^Target^ via electroporation and delivered into the fibrotic area (Fig. 2B). Lung macrophages treated with the siDrp1-loaded Exo^Target^ (Exo^Tx^) exhibited less mitochondria and increased mitochondrial length compared to the controls (Fig. 2C). As a result, the CD206-targeting exosomes significantly alleviated lung fibrosis in the bleomycin-induced mice (Fig. 2D). Complementing these biological delivery systems, nanostructured materials demonstrate dual capacity for mitochondrial morphology regulation and immunomodulation. Shanley et al. [136] observed that there was a trend toward an increase in mitochondrial aspect ratio and eccentricity upon nanosized hydroxyapatite (nanoHA) particle stimulation, with mitochondrial structures appearing as fused networks within the macrophages, resulting in anti-inflammatory macrophage polarization. Similarly, our group developed HA@Ce-TA nanoparticles (HA@Ce-TA NPs), which incorporate hydroxyapatite and tannic acid (TA) to target mitochondria without relying on mitochondrial membrane charge [137]. These nanoparticles positively modulate mitochondrial dynamics by regulating mitochondrial adenosine triphosphatase (ATPase)/ATP synthase-related signaling pathways. Notably, HA@Ce-TA NPs have been demonstrated to shift macrophage polarization from the pro-inflammatory M1 phenotype to the anti-inflammatory M2 phenotype by suppressing inflammatory cascades, thereby facilitating macrophage transition and promoting repair in bone defect models.

Emerging therapeutic modalities are extending beyond canonical mitochondrial dynamic regulation to target cristae architecture. This paradigm shift recognizes cristae remodeling as a potent immune-metabolic control point, enabling precise macrophage reprogramming through organellar nanoengineering. Among these, plant-derived nanovesicles (PDNVs) have garnered increasing interest due to their remarkable biocompatibility, low immunogenicity, cost-effectiveness, and ease of production. Nanovesicles derived from *Artemisia annua L.* (ADNVs), prepared through sequential ultracentrifugation, have demonstrated the ability to restore mitochondria damaged by LPS stimulation [138]. Specifically, ADNVs transform swollen, disorganized mitochondria with disrupted or absent cristae into elongated mitochondria with well-organized cristae. This mitochondrial restoration is mediated by γ-aminobutyric acid (GABA) present in ADNVs, which acts via GABA receptors, particularly GABABR. Improved mitochondrial morphology enhances mitochondrial function and reprograms macrophages toward the anti-inflammatory M2 phenotype. As a result, ADNVs enhance mitochondrial integrity and alveolar macrophage (AM) functionality, providing protection against acute lung injury (ALI) and mitigating lung immunopathology. In addition to plant-derived approaches, microorganisms can also influence mitochondrial structure. For instance, *Lactobacillus paracasei* KW3110, a specific lactic acid bacterial strain, can restore mitochondrial shape and cristae disrupted by LPS exposure [139]. By preserving cristae integrity, KW3110 alleviates mitochondrial dysfunction in inflammation-stressed macrophages. This preservation is crucial since cristae integrity supports mitochondrial respiration by expanding the inner membrane surface area, thereby accommodating essential respiratory protein complexes. Such microbial–mitochondrial interplay highlights the therapeutic potential of symbiotic mechanisms to mitigate pathological cristae remodeling. Consequently, both plant-derived and microbial-based platforms emerge as novel bioengineering strategies for immune-metabolic disorders.

### Mitophagy modulation: Enhancing clearance efficiency

**Fig. 3.**
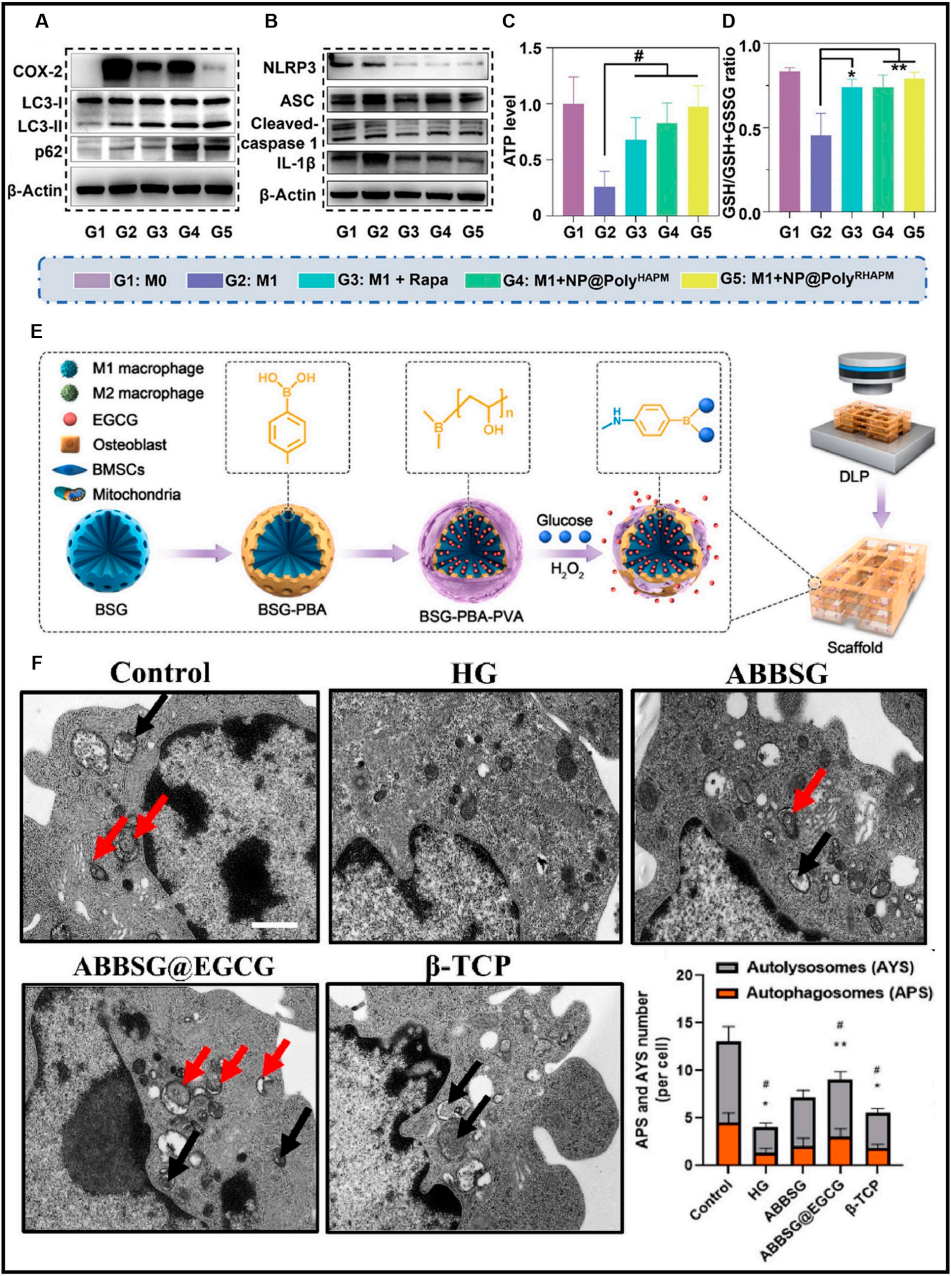
(A) NP@Poly^RHAPM^ alleviates the blockage of autophagic flow. (B) Autophagy limits the activation of NLRP3 inflammatory vesicles. (C) ATP production in M1 macrophages after NP@Poly^RHAPM^ treatment. (D) Macrophage responsiveness to oxidative stress after NP@Poly^RHAPM^ treatment [141]. Copyright 2023, Wiley-VCH GmbH. (E) Schematic illustration of the fabrication of glucose and hydrogen peroxide dual-responsive BSG scaffolds loaded with EGCG. (F) EGCG-loaded BSG markedly restores the number of autophagic vesicles in macrophages under high-glucose (HG) conditions [142]. Copyright 2024, KeAi.

Mitophagy has emerged as a master switch governing macrophage functional plasticity, driving the development of cutting-edge toolkit to spatiotemporally control its activity for immunotherapeutic applications. By coupling mitochondrial quality control with metabolic reprogramming, these approaches offer precision control over inflammatory resolution and tissue repair across diverse pathological contexts. Rapamycin (Rapa) is a powerful autophagy activator known for its marked anti-inflammatory properties, achieved through the induction of autophagic pathways [140]. However, its poor water solubility limits its bioavailability and delivery efficiency, particularly when targeting macrophages. This limitation underscores the pressing need for innovative delivery strategies that enhance the therapeutic efficacy of Rapa, minimize off-target drug accumulation, and mitigate potential side effects associated with its systemic distribution. Li et al. [141] developed a ROS-responsive biodegradable amphiphilic polymer (Poly^HAPM^), which was used to encapsulate Rapa into nanoparticles, forming NP@Poly^RHAPM^. This nanomaterial was designed for intra-articular injection to address osteoarthritis (OA). Treatment with NP@Poly^RHAPM^ significantly increased the LC3-II/I ratio and p62 protein expression in M1 macrophages compared to controls, indicating enhanced mitochondrial autophagy (Fig. 3A). Autophagic process inhibited the activation of NLRP3 inflammasomes, thereby preventing persistent inflammation (Fig. 3B). This process also facilitated energy production, maintained mitochondrial quality by eliminating damaged mitochondria, and reduced excessive ROS levels (Fig. 3C). Furthermore, they improved macrophages’ ability to withstand oxidative stress (Fig. 3D). Consequently, NP@Poly^RHAPM^ effectively suppressed M1 macrophage polarization and promoted their repolarization toward the anti-inflammatory M2 phenotype in synovial tissue. However, direct administration of this nanomaterial confronts challenges such as uncontrolled drug release and restricted applicability to ROS-elevated lesions. Furthermore, while these systems exhibit responsive behavior primarily in ROS-enriched environments, their targeting specificity and therapeutic efficacy require substantial enhancement. Tian and his team [142] designed an innovative dual-responsive borosilicate glass (BSG) scaffold capable of releasing epigallocatechin gallate (EGCG) in response to glucose and H_2_O_2_, targeting the dysregulated inflammation associated with diabetic alveolar bone defects. EGCG, a potent antioxidant and anti-inflammatory compound primarily found in green tea, has limited bioavailability when administered orally. To overcome this, the researchers incorporated EGCG into the mesoporous structure of BSG through physical adsorption, followed by encapsulation with polyvinyl alcohol to regulate its release (Fig. 3E). Under conditions of chronic hyperglycemia or elevated ROS, the scaffold triggers EGCG release, promoting autophagy in macrophages, relieving autophagic flux blockages, and restoring mitochondrial function (Fig. 3F). This controlled delivery system effectively reprograms inflammatory macrophages into an anti-inflammatory phenotype, offering a targeted approach for managing inflammation in diabetic high-glucose environments. In addition to artificial materials, organism-derived substances can be used to modulate mitochondrial autophagy. Conidiogenone C is a natural cyclopiane diterpenoid derived from marine microorganisms. Li et al. [143] identified conidiogenone C as a small-molecule activator of immunity-related GTPase family M protein 1 (IRGM1), marking the first reported activator of this protein. Their study demonstrated that conidiogenone C directly binds to IRGM1 in LPS-stimulated RAW264.7 macrophages, thereby promoting IRGM1-mediated mitophagy of dysfunctional mitochondria. This process facilitates the clearance of cytosolic mtDNA and subsequently suppresses IFN responses in pro-inflammatory macrophages. Beyond conidiogenone C, other natural compounds have also been found to regulate mitophagy and influence immune responses. Alliin, an organosulfur compound extracted from garlic, has high therapeutic and pharmacological properties. Liu et al. [144] demonstrated that alliin could induce the alleviating effect of pyroptosis by promoting mitophagy in LPS-induced THP-1 macrophages and mice, as it is mostly caused by mtROS from dysfunctional mitochondria. Most therapeutic strategies engage in mitochondrial quality control by promoting mitophagy, thereby inhibiting macrophage pro-inflammatory polarization. However, some studies have also achieved this by inhibiting mitophagy. Meng et al. [145] demonstrated that taurine inhibits S-adenosylmethionine (SAM)-dependent PP2Ac methylation to block PINK1-mediated mitophagy flux, thereby maintaining a high mitochondrial density, which ultimately reprograms the polarized phenotype of macrophages by hindering the conversion of energy metabolism to glycolysis required for M1. Such a paradox may stem from the dynamic equilibrium nature of mitochondrial quality control, where both hyperactivated and suppressed autophagic flux can destabilize this balance and impair macrophage signaling homeostasis. Collectively, pharmacologic, bioengineering, and natural compound-based approaches for bidirectionally modulating mitophagic flux establish a powerful paradigm for macrophage fate reprogramming across the inflammatory disease spectrum. Although current strategies show therapeutic potential, their clinical translation remains constrained by spatiotemporal and microenvironmental limitations. Next-generation integration of microenvironment-responsive delivery with multi-target strategies may unlock precision mitophagy control for macrophage reprogramming across disease states.

Ultrasound (US)-responsive piezoelectric nanomaterials represent a novel and promising approach for precise intracellular modulation. Wang and his group [146] developed a piezoelectric nanosystem that enables precise and noninvasive regulation of mitochondrial autophagy through US stimulation. The system, named BaTCG, consists of barium titanate nanoparticles modified with triphenylphosphine (TPP) for mitochondrial targeting and loaded with GLP-1 receptor agonists. Upon US stimulation, the piezoelectric effect induces localized currents that depolarize the mitochondrial membrane and activate mitophagy. Compared with traditional approaches using chemical inducers or uncouplers, this strategy provides better spatial and temporal control and reduces systemic toxicity. After entering cells, the nanoparticles accumulate near mitochondria, and mitophagy is triggered only when US is applied, allowing precise control over the treatment site and timing. This approach promotes the clearance of dysfunctional mitochondria and relieves oxidative stress at its source, overcoming the limitations of conventional antioxidant therapies. Although originally applied to diabetes-related erectile dysfunction, the method offers a generalizable strategy for mitochondrial quality control. The study highlights the potential of US-responsive piezoelectric nanomaterials for targeted intracellular modulation and provides a new avenue for regulating macrophages through mitochondrial autophagy.

Mitophagy serves as a bidirectional regulator of macrophage polarization. Zhang et al. [147] demonstrated that polyethylene glycol-conjugated gold nanoparticles (PEG-AuNPs) can effectively modulate mitophagy in tumor-associated macrophages (TAMs), promoting their repolarization from the M2 phenotype to the M1 phenotype. Specifically, PEG-AuNPs induce lysosomal alkalization, disrupting the acidic environment typically required for efficient mitochondrial degradation. Furthermore, PEG-AuNPs enhance lysosomal membrane permeability, impairing the fusion of autophagosomes with lysosomes and preventing the complete degradation of engulfed mitochondria. By interfering with autophagic flux, PEG-AuNPs hinder the polarization of TAMs toward the M2 phenotype, which is often associated with tumor-promoting and immunosuppressive functions. In contrast, inhibition of mitochondrial autophagy favors the repolarization of TAMs toward the M1 phenotype, enhancing antitumor immunity and supporting the potential for effective cancer immunotherapy.

### Biogenesis stimulation: Boosting organelle formation

Targeted induction of mitochondrial biogenesis represents a promising therapeutic strategy for macrophage functional reprogramming. This approach capitalizes on the intrinsic cellular capacity of de novo mitochondrial biosynthesis to reestablish metabolic equilibrium, thereby modulating polarization states and inflammatory output. Current advances employ diverse modalities, ranging from natural bioactive components to engineered synthetic systems to amplify PGC-1α-driven transcriptional networks, thereby coupling mitochondrial rejuvenation with immunomodulation [148,149].

Medicinal PDNVs have garnered considerable interest for modulating mitochondrial biogenesis. Ye et al. [138] recently demonstrated that treatment with ADNVs significantly improved the expression of TFAM, a nuclear-encoded transcription factor essential for mtDNA replication and stability, and PGC-1α, a key transcriptional coactivator of TFAM, in LPS-stimulated macrophages. These findings suggest that ADNVs enhance mitochondrial biogenesis, promote OXPHOS activity, boost ATP production, and restore redox balance in dysfunctional macrophages. Sharing botanical origins, TQC induces similar functional recovery in mitochondrial biogenesis [134]. However, in contrast to the transcriptional mechanisms, TQC pretreatment exerts mitochondria-protective effects primarily through potent induction of HO-1. This antioxidant enzyme promotes rapid recovery of mitochondrial biogenesis in LPS-activated macrophages by restoring ATP synthesis capacity and neutralizing mtROS, without engaging PGC-1α/NRF1/TFAM-dependent pathways.

Complementary to plant-derived strategies, synthetic biomaterials offer precision-controlled activation of mitochondrial biogenesis. The biomimetic carbon monoxide (CO) nanogenerator (M/PCOD@PLGA) enhances PGC-1α signaling through multiple mechanisms linked to its controlled CO release and peroxynitrite (ONOO^−^) scavenging [150]. CO directly activates the AMPK/SIRT1 pathway, which deacetylates and activates PGC-1α, a master regulator of mitochondrial biogenesis. Simultaneously, the elimination of cytotoxic ONOO^−^ by PCOD585 reduces oxidative stress-mediated suppression of PGC-1α transcriptional activity. CO further potentiates mitochondrial biogenesis by up-regulating Nrf1 and TFAM, downstream targets of PGC-1α. This dual action—AMPK/SIRT1 activation coupled with redox homeostasis restoration—synergistically amplifies PGC-1α-driven mitochondrial DNA replication and respiratory chain enzyme synthesis, thereby supporting macrophage metabolic reprogramming toward an anti-inflammatory phenotype.

While mitochondrial biogenesis represents an endogenous process requiring sustained activation to exert therapeutic effects, mitochondrial transplantation offers a promising complementary strategy capable of providing immediate metabolic rescue to distressed cells. The biomimetic a127/mito@ZIF@Ma nanoplatform utilizes exogenously isolated healthy mitochondria from donor cells, which are conjugated with macrophage membrane-camouflaged ZIF-8 nanoparticles carrying anti-miR-127 [151]. This system directly delivers functional mitochondria to recipient macrophages while simultaneously triggering endogenous mitochondrial biogenesis through epigenetic modulation. Unlike conventional approaches that solely rely on pharmacological activation of mitochondrial synthesis pathways, this dual-action strategy provides immediate metabolic rescue via transplanted organelles while durably enhancing self-renewal capacity through PGC-1α pathway stimulation. The combined effect overcomes the kinetic limitations of purely endogenous biogenesis methods, resulting in accelerated metabolic recovery characterized by restored OXPHOS and rebalanced redox homeostasis. Consequently, macrophages exhibit a phenotypic shift toward pro-resolving M2 states with reduced inflammatory cytokine secretion and enhanced tissue repair functions. Although challenges regarding mitochondrial sourcing scalability persist, this approach demonstrates superior efficacy in acute inflammatory settings where rapid metabolic correction is critical.

### Metabolic reprogramming: Optimizing energy flow

**Fig. 4.**
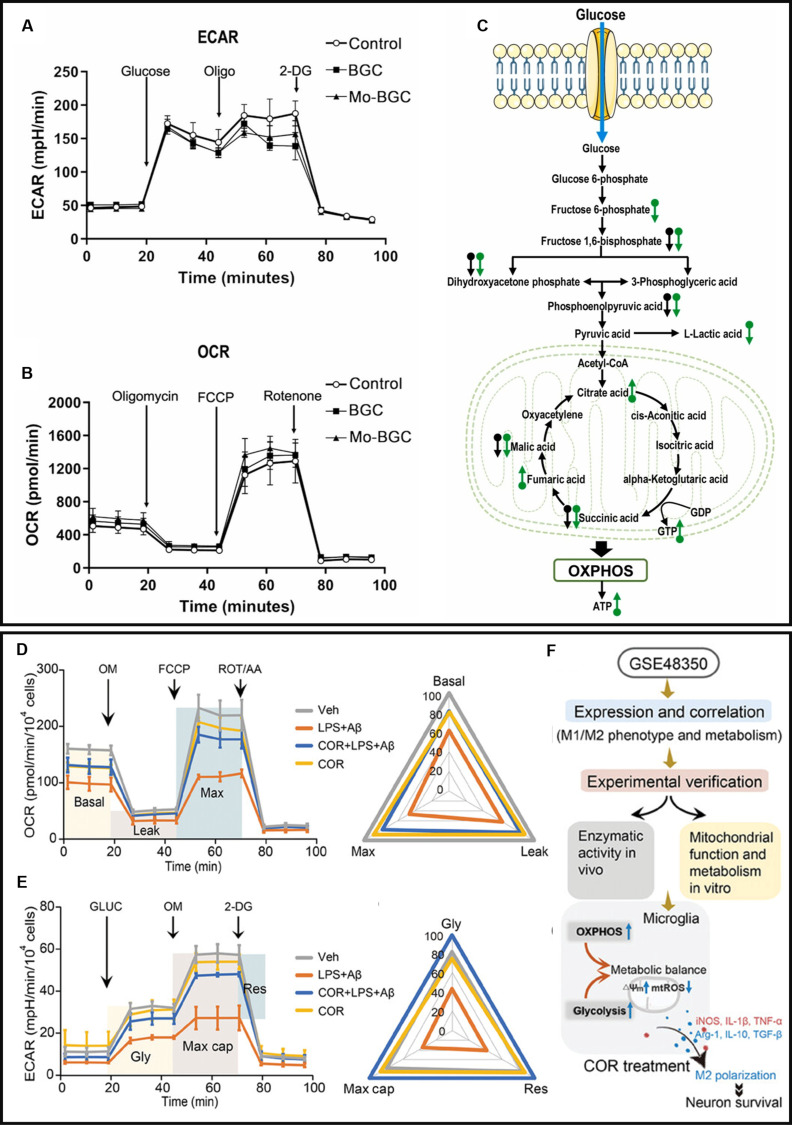
(A) ECAR level of macrophages in response to Mo-BGC. (B) OCR level of macrophages in response to Mo-BGC. (C) Mo-BGC enhances TCA cycle metabolites and OXPHOS while attenuating glycolysis of macrophages [154]. Copyright 2024, Elsevier. (D) OCR level of microglia in response to COR. (E) ECAR level of microglia in response to COR. (F) Both OXPHOS and glycolysis are up-regulated to maintain mitochondrial homeostasis in AD microglia following COR treatment. Copyright 2024, Wiley-VCH GmbH.

Mitochondrial metabolism functions as a central regulator of macrophage functional reprogramming, providing a foundation for developing context-specific therapeutic strategies. By harnessing bioengineered materials, organelle-precise delivery systems, and natural/synthetic modulators, researchers now aim to recalibrate metabolic nodes spanning electron transport efficiency, metabolite flux, and redox balance. These approaches, though varied in design, converge on a unified goal: spatiotemporal control over macrophage polarization through mitochondrial bioenergetic rewiring, offering transformative potential for immune-metabolic disease intervention. Molybdenum (Mo), an essential trace element for nearly all organisms, has been identified as part of the active sites of more than 50 enzymes involved in various redox reactions and oxygen atom transfer [152]. Huang et al. [153] engineered a pH-responsive molybdate-oligosaccharide nanoplatform (CMO NPs) that orchestrates diabetic wound healing through dual immune-metabolic modulation. By up-regulating SIRT1–PGC-1α axis components and respiratory complex subunits, CMO NPs enhance bioenergetic efficiency, driving macrophage M2 polarization via augmented OXPHOS capacity—a metabolic shift that concurrently dampens inflammatory cascades. However, the pH-dependent activation of CMO NPs limits their applicability to other microenvironments; future iterations integrating multi-omic metabolic maps could expand their operational range across heterogeneous disease niches. Building upon this metabolic paradigm, He et al. [154] developed molybdenum-doped bioactive scaffolds (Mo-BGC) that induce dose-dependent metabolic rewiring in periodontal macrophages. Glycolytic profiling revealed that Mo-BGC treatment markedly attenuated macrophage glycolytic capacity and reserve relative to controls, whereas BGC induced only a marginal reduction in glycolytic reserve (Fig. 4A). Mitochondrial functional analysis showed that Mo-BGC treatment robustly enhanced basal respiration, maximal respiration, and ATP production in macrophages compared to both BGC and control groups (Fig. 4B). Following incubation with Mo-BGC powder extract, macrophages exhibited significant up-regulation of pivotal mitochondrial metabolites including fumarate, guanosine triphosphate (GTP), citrate, and ATP. This metabolic reprogramming was accompanied by enhanced mitochondrial bioenergetics, which mechanistically drove the M2 polarization phenotype through improved mitochondrial membrane potential and OXPHOS efficiency (Fig. 4C). Additionally, the precisely printed hollow-pipe structure facilitated large and sustained release of bioactive ions from the Mo-BGC scaffolds, allowing this biomaterial to exert long-term effects in vivo. Building upon bioactive glass ceramic matrices, Chen et al. [155] engineered selenium-doped (Se-BGC) constructs that simultaneously drive macrophage M2 polarization and osteogenic metabolism reprogramming. Its enhancement of the macrophage OXPHOS metabolism contributed to macrophage polarization regulation toward M2. Despite their metabolic efficacy, such bulk material approaches exhibit limited organelle-level precision, often inadequately resolving the underlying bioenergetic disturbances driving mitochondrial dysfunction. Addressing this therapeutic gap, Ren’s team [156] created mitochondrial complex biomimetic nanozymes (MCBN) through MnO₂ nanoarchitecture engineering. MCBN specifically targets mitochondria, where it absorbs excess electrons from the ETC, thereby restoring normal electron transfer and utilizing these electrons to decompose H_2_O_2_, transforming it from a harmful to a beneficial molecule. Experimental validation demonstrated that MCBN stabilizes the ETC and reduces ROS levels, thereby modulating NLRP3 inflammasome activation and the NF-κB signaling pathway. This mechanism effectively suppresses macrophage-driven inflammatory activation, providing therapeutic potential for periodontitis by controlling inflammation and alleviating alveolar bone resorption.

Natural bioactive compounds have been extensively studied for their potential in immunomodulation and regulation of mitochondrial metabolism. Macrophage migration inhibitory factor (MIF), an enigmatic tautomerase, was discovered to regulate M1 polarization. Vámos et al. [157] reveal that KRP-6, a potent and highly selective MIF ketonase inhibitor, improved mitochondrial bioenergetics by ameliorating basal respiration, ATP production, coupling efficiency, and maximal respiration in LPS + IFN-γ-treated cells. KRP-6 also reduced glycolytic flux in M1 macrophages, significantly reducing macrophage activation. Cordycepin (3′-deoxyadenosine, COR) is the first nucleoside antibiotic isolated from fungi. Zhong and his team [158,159] discovered that COR promotes M2 polarization of microglia by modulating mitochondrial metabolism, thereby improving the neuronal microenvironment in Alzheimer’s disease (AD). LPS + amyloid-β (Aβ) disrupted both oxidative metabolism (Fig. 4D) and glycolytic function (Fig. 4E) in microglia, with COR combination treatment uniquely reversing these metabolic deficits, aligning with its role in mitigating AD-related mitochondrial dysfunction. Specifically, COR targets hexokinase II (HKII) to enhance extracellular acidification rate (ECAR) and lactate production in the glycolytic pathway while simultaneously up-regulating oxygen consumption rate (OCR) in the OXPHOS pathway via pyruvate dehydrogenase kinase 2 (PDK2)-mediated activation of PDH (Fig. 4F). Sulforaphane, an organosulfur compound found as glucosinolate precursor in cruciferous vegetables including broccoli, Brussels sprouts, and cabbage, has been reported to influence mitochondrial metabolism reprogramming during M1 polarization, as well as contribute to inhibition of M1 marker expression in murine macrophages [160]. Grossamide (GSE), a lignanamide from *Polygonum multiflorum* Thunb., shunted NO production from arginine by up-regulation of arginase and down-regulation of inducible NO synthase, thus attenuating the inhibition of NO on OXPHOS and significantly inhibiting the release of IL-1β and TNF-α on LPS-induced macrophages [161]. Although GSE’s NO-centric mechanism effectively targets specific inflammatory pathways, its limited capacity to resolve broader metabolic dysregulation could be addressed through integration with mitochondrial metabolite sensors, enabling the construction of feedback-controlled therapeutic systems.

Currently, many strategies for macrophage modulation explore chemical drugs conjugated to various functional nanocarriers recognized by macrophage receptors. Metal–organic supercontainers (MOSCs) are self-assembled nanocarriers with multiple binding sites and good biocompatibility, ideal for drug delivery. Chen et al. [162] developed a zinc-based MOSC (Zn-NH-pyr) that efficiently targets mitochondria by escaping endo/lysosomal barriers. They loaded 4-octyl itaconate (4-OI) into this system to create 4-OI@Zn-NH-pyr. As expected, due to the highly efficient mitochondrial targeting efficacy, 4-OI@Zn-NH-pyr exhibited an enhanced inhibitory effect on basal respiration, ATP production, and maximal respiration, in comparison with the single 4-OI-treated group. However, the use of these targeting nanocarriers is usually limited by high cost, instability, toxic effects, and so on. Therefore, it is essential to design and develop structure-inherent small molecules targeting the mitochondria of macrophages. Wang et al. [163] screen the dyes and characterize a small-molecule infrared dye termed IR-61, which preferentially accumulates in the mitochondria of these pro-inflammatory adipose tissue macrophages. It improves obesity and its associated metabolic syndromes via intraperitoneal administration, potentiates mitochondrial OXPHOS, and thus represents a possible treatment strategy for obesity-related metabolic diseases. IR-61’s empirical screening is labor-intensive and serendipity-driven, and artificial intelligence (AI)-guided predictions of structure–mitochondrial affinity could accelerate the discovery of next-generation dyes with enhanced selectivity and metabolic reprogramming precision. These pioneering efforts exemplify the potential of mitochondrial metabolic engineering to steer macrophage fate, yet key challenges persist. Future progress will require multi-omic metabolic profiling to transcend microenvironmental constraints, feedback-controlled systems integrating metabolite sensors, and AI-driven platforms to deconvolute structure–mitochondrial affinity relationships. Through the application of these new technologies, next-generation strategies will achieve context-adaptive precision, transforming mitochondrial metabolism into a dynamically programmable lever for macrophage immunomodulation.

**Fig. 5.**
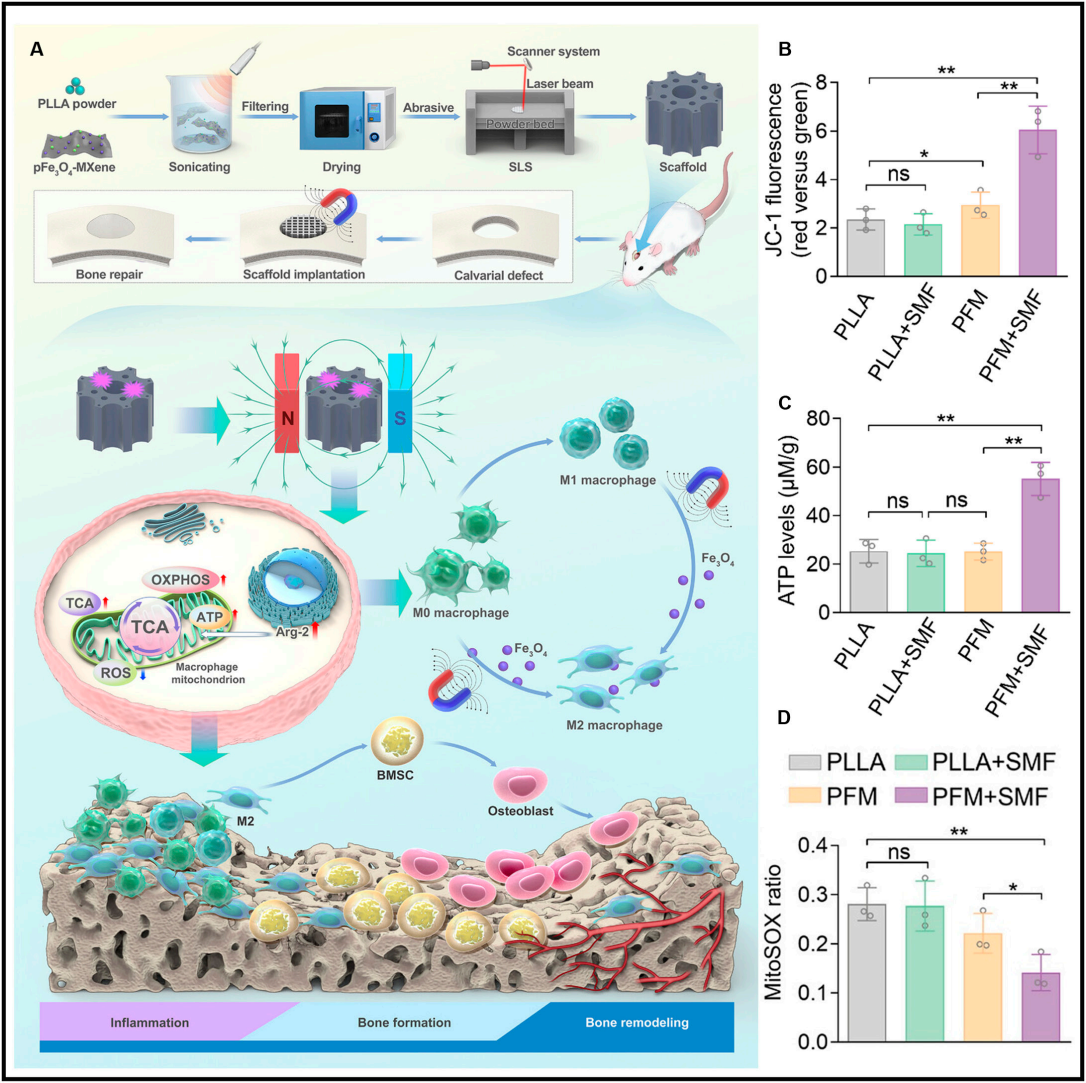
(A) Scheme showing the fabrication of magnetically responsive scaffold system and its SMF-induced mitochondrial modulation in macrophages. (B) Increased mitochondrial membrane potential in the PFM + SMF group. (C) Elevated intracellular ATP levels observed in the PFM + SMF group. (D) mtROS levels were reduced by the PFM scaffold under SMF intervention [164]. Copyright 2025, Wiley-VCH GmbH.

Conventional biomaterials designed to modulate macrophage polarization typically depend on passive structural properties or localized release of biochemical signals. These approaches exhibit limited spatiotemporal control and minimal influence on mitochondrial metabolic regulation. In comparison, magnetically responsive platforms permit noninvasive remote manipulation of cellular functions via externally applied magnetic fields, offering unprecedented precision in immune cell programming. A recent study by Yan et al. [164] introduced a magnetically responsive scaffold system that reprograms macrophage metabolism and polarization via targeted mitochondrial modulation (Fig. 5A). The authors engineered a nanocomposite by electrostatically assembling positively charged Fe_3_O_4_ (pFe_3_O_4_) nanoparticles onto 2-dimensional MXene nanosheets, creating a co-dispersed hybrid that was embedded into a poly-l-lactic acid (PLLA) matrix through selective laser sintering to fabricate a biomimetic porous bone scaffold (PFM). This structure displayed excellent mechanical properties, biocompatibility, and magnetic sensitivity. Upon exposure to a static magnetic field (SMF), the PFM scaffold significantly elevated the expression of arginase 2 (Arg2), a mitochondrial enzyme that modulates oxidative metabolism. Subsequent functional assays showed that Arg2 up-regulation was accompanied by increased mitochondrial membrane potential, as indicated by higher JC-1 aggregate formation in the PFM + SMF group (Fig. 5B). This increase in membrane potential correlated with enhanced ATP production (Fig. 5C). Meanwhile, mtROS levels were significantly reduced, suggesting improved mitochondrial efficiency and reduced oxidative stress (Fig. 5D). This metabolic shift promoted macrophage polarization toward the anti-inflammatory M2 phenotype, contributing to a favorable immune microenvironment that attenuated early inflammatory responses and supported bone regeneration and neovascularization. This work highlights a novel strategy that integrates advanced material design with remote magnetic control to achieve precise regulation of mitochondrial metabolism. By leveraging the interplay between biophysical cues and mitochondrial metabolism, magnetically responsive scaffolds offer distinct advantages over conventional biomaterials in orchestrating immune responses and enhancing tissue repair, positioning them as promising platforms for next-generation regenerative therapies.

Material-based strategies for modulating mitochondrial function have traditionally faced limitations such as indirect regulation, low specificity, and delayed cellular responses, which are effectively overcome by directly delivering exogenous mitochondria. Zhang et al. [165] demonstrated such advancement by employing a targeted mitochondrial transplantation system to reprogram TAMs toward a pro-inflammatory M1-like phenotype, thereby enhancing anti-tumor immune responses. This system utilizes mannosylated polyethyleneimine (mPEI) to coat mitochondria isolated from M1 macrophages, facilitating selective uptake by M2 macrophages through mannose receptor-mediated endocytosis. Upon internalization, the exogenous mitochondria shift cellular metabolism from OXPHOS to glycolysis, as demonstrated by increased ECARs and decreased oxygen consumption. This metabolic reprogramming elevates intracellular ROS, which activates key signaling pathways including NF-κB and MAPK, promoting and stabilizing the inflammatory M1-like immunophenotype. The successful mitochondrial transplantation strategy not only achieves precise metabolic reprogramming of macrophage mitochondria but also provides crucial theoretical and practical insights for directly modulating cellular metabolic states via active organelles, advancing the field of mitochondrial metabolic regulation. Despite its therapeutic potential, mitochondrial transplantation faces translational challenges in clinical research. Numerous studies have emphasized that the inherent fragility of isolated mitochondria necessitates rapid processing under strictly controlled low-temperature conditions to preserve viability and cannot be stored permanently. These biological limitations underscore the critical need to ensure sustained mitochondrial functionality, stability, and therapeutic efficacy for human applications.

### ROS scavenging: Limiting oxidative stress

Mitochondrial bioenergetic activity constitutes the primary source of cellular ROS, with redox disequilibrium between pro-oxidant generation and antioxidant buffering capacity posing a fundamental threat to organellar homeostasis. This delicate redox equilibrium is dynamically maintained by enzymatic and nonenzymatic antioxidant systems that not only neutralize ROS overaccumulation but also modulate oxidative signaling cascades governing macrophage phenotypic transitions.

**Fig. 6.**
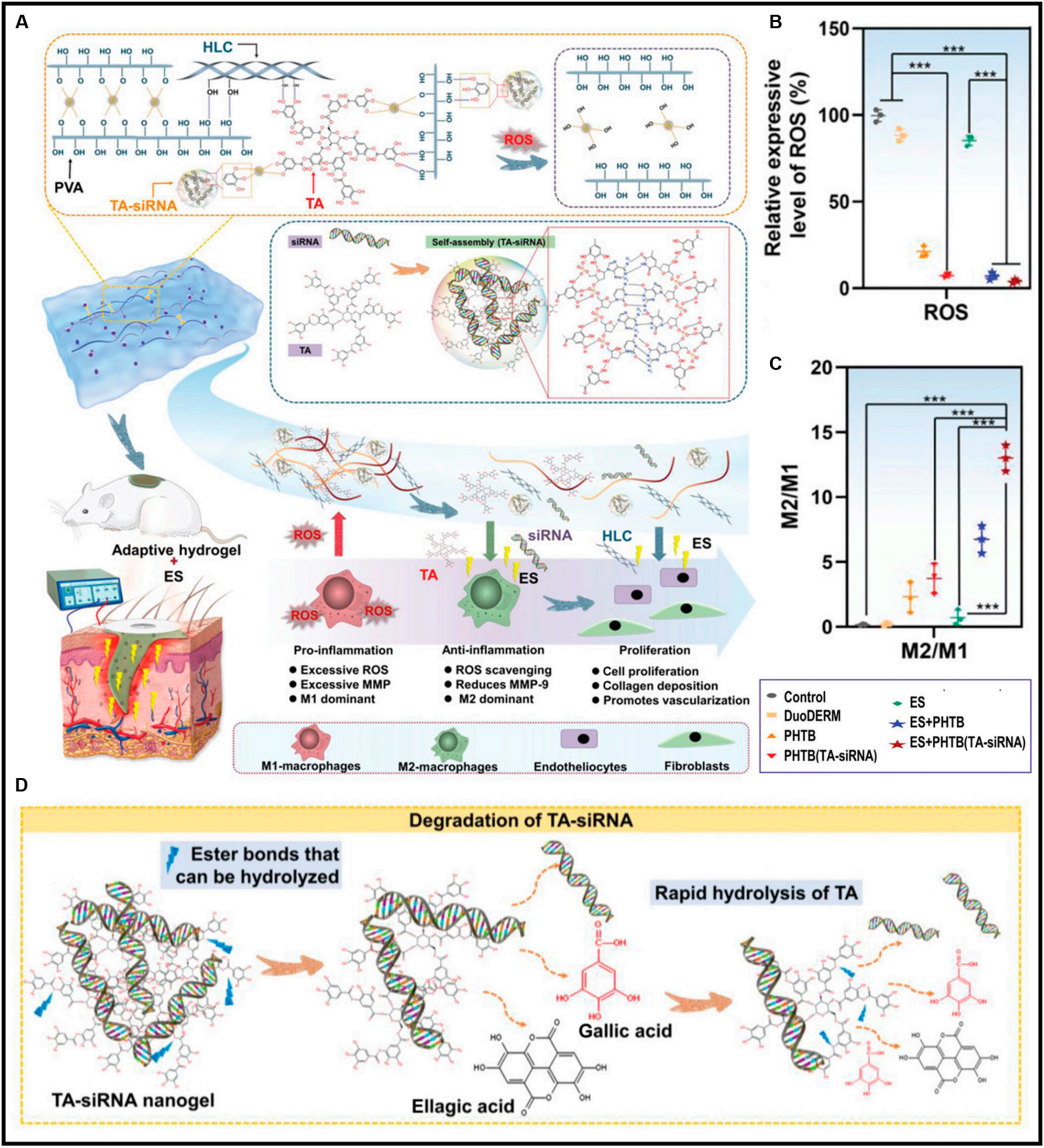
(A) Schematic illustration of TA-siRNA nanogel synthesis and their dual therapeutic action. (B) TA in the PHTB(TA-siRNA) hydrogel effectively reduces ROS levels in diabetic chronic wounds, as compared to the control groups. (C) The M2/M1 macrophage ratio in the ES therapy–PHTB(TA-siRNA) hydrogel groups was significantly higher than in the other groups. (D) Mechanism of siRNA release from TA-siRNA nanogels [168]. Copyright 2022, Wiley-VCH GmbH.

Research has shown that various natural products, including polyphenols like TA, are widely used as anti-inflammatory agents and antioxidants due to their notable ROS-scavenging properties [166]. Li and his team [167] reported the development of a TA-based DDS that degrades in response to ROS due to its sensitivity to H_2_O_2_. Upon the breakdown of the DDS, TA is released, facilitating the removal of excess ROS. The combined delivery of anti-inflammatory drugs and ROS elimination effectively promotes macrophage repolarization during inflammation. Building on previous reports of TA-based ROS-responsive DDSs, Lei and Fan [168] advanced this concept by developing PHTB (TA-siRNA) hydrogels, where TA serves dual roles as both a nanogel scaffold and bioactive agent (Fig. 6A). The catechol-rich structure enables effective ROS neutralization, disrupting oxidative stress cycles that sustain pro-inflammatory M1 macrophage dominance (Fig. 6B). This antioxidant activity synergizes with small interfering RNA (siRNA)-mediated matrix metalloproteinase-9 (MMP-9) silencing and electrical stimulation (ES)-enhanced delivery to promote M2 macrophage polarization, as evidenced by an increase in the M2/M1 ratio (Fig. 6C). In this work, TA-siRNA nanogels were engineered to leverage TA’s ROS responsiveness for controlled therapeutic release, utilizing the hydrolysis of ester bonds within the TA structure to trigger degradation-dependent siRNA delivery (Fig. 6D). This design not only ensures controlled siRNA release but also minimizes off-target effects, enhancing the precision of siRNA-mediated gene silencing in macrophages. These findings establish TA as a versatile therapeutic component for engineering multifunctional platforms targeting redox-imbalanced chronic inflammation. In addition to polyphenols, compounds such as flavonoids [169], spherical melanin nanoparticles (MNPs) extracted from cuttlefish ink [170], and astaxanthin [141], a naturally occurring fat-soluble carotenoid, have been shown to exhibit free radical scavenging and antioxidant effects. These compounds have also been investigated for their delivery to key sites to modulate macrophage polarization phenotypes for disease treatment.

**Fig. 7.**
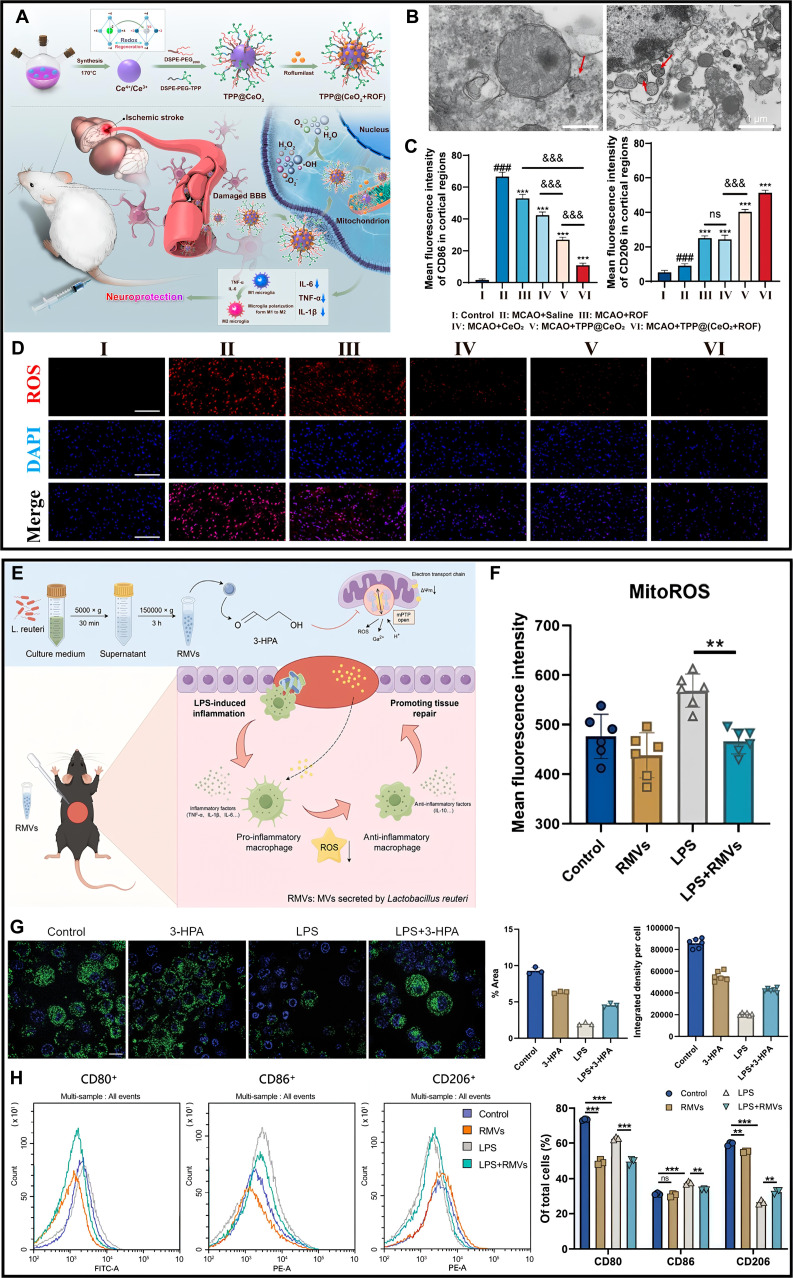
(A) Schematic diagram of intravenous TPP@(CeO_2_+ROF) targeting mitochondria to release drugs, exerting protective effects against ischemic stroke. (B) Electron microscopy images of TPP@(CeO_2_+ROF) aggregation in mitochondria. (C) Mitosox Red fluorescent probe was used to assess mitochondrial ROS levels following treatment with each experimental group. (D) TPP@(CeO_2_+ROF) treatment modulated CD86 and CD206 expression in microglia [180]. Copyright 2024, American Chemical Society. (E) Schematic diagram demonstrating how RMVs modulate oxidative stress in activated macrophages to aid wound healing. (F) RMV normalizes mtROS levels in LPS-affected macrophages. (G) 3-HPA reverses the impact of LPS on mitochondrial permeability in macrophages. (H) RMV reverses the classical macrophage activation pathway promoted by LPS [182]. Copyright 2024, Wiley-VCH GmbH.

The ROS detoxification system is composed of natural enzymes such as superoxide dismutase (SOD) [171], catalase (CAT) [172], GSH peroxidase, and GSH reductase [172]. Oxygen radicals are converted into H_2_O_2_ by SOD, and H_2_O_2_ is converted into water by CAT or GSH peroxidase for the purpose of detoxification. In recent years, materials with catalytic ROS-scavenging activity have also been found to be alternative or even more effective strategies for regulating macrophages’ fate, attributed to their enzyme-like behavior [173]. A series of nanomaterials such as cerium oxide, MnO_2_-x, Fe_3_O_4_, Prussian blue, and their composites have good antioxidant-like enzyme activity with higher stability than natural enzymes [174,175]. Catalytic nanoparticles (NPs), such as manganese ferrite NPs, have recently been developed as biocompatible therapeutic agents capable of continuous oxygen generation in H₂O₂-rich hypoxic environments [176]. However, excessive concentrations of manganese ferrite NPs can lead to cytotoxicity and decreased oxygen production efficiency due to the generation of •OH as intermediates in the Fenton reaction. Kim’s group [177] demonstrated that ceria NPs can convert •OH into O₂ molecules. They further developed manganese ferrite and ceria nanoparticle-anchored mesoporous silica nanoparticles (MSNs), which synergistically scavenge ROS and generate oxygen, thereby reducing M1 macrophage levels and promoting M2 macrophage polarization for inflammation treatment. The redox-active surface of cerium oxide nanoparticles (CeO₂), characterized by abundant oxygen vacancies and dynamic Ce^3+^/Ce^4+^ transitions, underlies its dual enzyme-mimetic capacity to scavenge ROS. Nano-CeO₂ can mitigate local inflammatory responses by scavenging excessive ROS and suppressing M1 macrophage polarization. Nevertheless, the more important approach for treating inflammation is to activate macrophage polarization toward the M2 phenotype, and the M2 polarization function of CeO₂ is quite limited. Wang and his team [178] addressed this limitation by bonding quercetin, a natural antioxidant flavonoid, to CeO₂, resulting in superior antioxidant and anti-inflammatory properties. They further demonstrated that post-subgingival injection of this composite efficiently eliminated excessive ROS and reduced local periodontal inflammation in a rat model. Although CeO₂-mediated ROS scavenging has a limited direct impact on macrophage polarization itself, it can amplify the paracrine regulation of M2 polarization exerted by surrounding cells. Supporting this concept, Zheng et al. [179] incorporated cerium oxide nanoparticles (COPs) into polycaprolactone porous nanoscaffolds to develop the NS@COP scaffold for the treatment of traumatic spinal cord injury. Their findings demonstrate that the ROS-scavenging capability of these nanoparticles restores the responsiveness of pro-inflammatory macrophages to paracrine calcitonin gene-related peptide (CGRP) signals, primarily released by surrounding sensory neurons. This restoration occurs through the up-regulation of receptor activity modifying protein 1 (RAMP1), a vital component of the CGRP receptor. Consequently, the reinstated CGRP signaling promotes macrophage commitment to an anti-inflammatory M2 phenotype, thereby reducing glial scar formation. This approach pioneers a targeted strategy for modulating neuroimmune communication in spinal cord injury. However, this ROS-scavenging approach lacks mitochondrial targeting, compromising its therapeutic precision. Concurrently, advanced CeO₂ delivery systems designed for nervous tissue now incorporate mitochondrial targeting, demonstrating enhanced spatial control of ROS modulation (Fig. 7A) [180]. The TPP@(CeO₂+ROF) platform, functionalized with TPP, achieves selective accumulation in mitochondria as the primary site of ROS generation (Fig. 7B). As expected, TPP@(CeO_2_+ROF) demonstrates potent ROS-scavenging capabilities, effectively modulating oxidative stress levels within mitochondria through Ce^3+^/Ce^4+^ redox cycling (Fig. 7D). This nanosystem synergizes catalytic ROS decomposition with roflumilast (ROF)-mediated PDE4 inhibition, reducing neuronal apoptosis in vitro and cerebral infarct volume in vivo in stroke models. By directly quenching mtROS and restoring macrophage–neuron crosstalk, it amplifies M2 polarization, demonstrating spatially optimized immunomodulation (Fig. 7C). While current evidence is limited to indirect macrophage effects, these developments establish critical engineering principles for future systems that directly modulate neuroimmune macrophages through precision ROS scavenging. These findings collectively establish CeO₂ as a versatile platform for inflammatory regulation, where material hybridization and targeted delivery strategies synergistically bridge ROS scavenging with immune-metabolic reprogramming.

In addition to scavenging mtROS, several researchers tried to inhibit the generation of mtROS. Fan et al. [181] uncovered for the first time that down-regulated murine homolog of steatohepatitis-associated circRNA ATP5B regulator (circRNA mSCAR) promotes the development of sepsis, which correlates with the excessive M1 polarization. Similarly, they encapsulated exosomes with the therapeutic circRNA mSCAR followed by TPP–poly-d-Lysine (PDL) electroporation, achieving mitochondrial delivery of circRNA mSCAR. Precise delivery of circRNA mSCAR into mitochondrial could robustly reverse M1 polarization by reducing mtROS, and thus improve the outcome of septic condition, emerging as a promising intervening strategy of sepsis and other inflammatory diseases. Chen et al. [162] developed a zinc-based MOSC, Zn-NH-pyr, which exhibited highly efficient ROS-scavenging capacity and strong mitochondria-targeting ability. After encapsulating 4-OI, the resulting composite not only scavenged ROS exogenously but also reduced endogenous ROS production by modulating mitochondrial respiration in inflammatory macrophages. The endogenous reduction of ROS was primarily attributed to 4-OI, which activated Nrf2 to up-regulate endogenous antioxidant production and inhibited SDH to suppress mtROS production. Consequently, 4-OI@Zn-NH-pyr demonstrated marked efficacy in ameliorating joint inflammation by modulating macrophage activity. Additionally, increased mitochondrial permeability leads to the disassembly of respiratory supercomplexes and ETC dysfunction, resulting in a marked increase in ROS production. Chen et al. [182] identified that 3-hydroxypropionaldehyde (3-HPA), encapsulated within membrane vesicles secreted by *Lactobacillus reuteri* (RMVs), an endogenous human symbiont, inhibits LPS-induced macrophage polarization toward a pro-inflammatory phenotype (Fig. 7E). This effect arises from RMV-mediated regulation of mitochondrial complexes I and II, which stabilizes the LPS-disrupted mitochondrial membrane potential and consequently reduces mtROS production (Fig. 7F). Specifically, 3-HPA present in RMVs prevents LPS-triggered opening of the mPTP, thereby modulating mitochondrial permeability (Fig. 7G). As a result, RMVs attenuate pro-inflammatory macrophage polarization, mitigate the inflammatory microenvironment in wound tissues, and significantly enhance mucosal and skin wound healing (Fig. 7H).

While lowering mtROS promotes M2 macrophage polarization and tissue repair, in bacterial infections, a controlled increase of mtROS is crucial for activating macrophage antimicrobial functions. Controlled elevation of mtROS activates macrophages to kill pathogens like *Staphylococcus aureus*, which evade immunity by suppressing macrophage function. Conventional methods to enhance ROS rely on catalytic nanoparticles requiring exogenous substrates like H_2_O_2_ and pose potential risks due to uncontrolled and persistent catalytic reactions. In contrast, Feng et al. [183] developed piezocatalytic nanoparticles (piezoNPs) to generate ROS under US without chemical substrates, allowing precise temporal and spatial control. Upon US stimulation, piezoNPs convert mechanical energy into surface charge that reduces oxygen to superoxide, mimicking endogenous mtROS production. To ensure cytosolic activation, piezoNPs are coated with a metal-phenolic network (MPN), which prevents ROS generation in extracellular environments and enables lysosomal escape via proton buffering. Once in the cytoplasm, US triggers on-demand ROS production, reprogramming macrophages to an activated state with enhanced bactericidal capacity. Compared to traditional approaches, this method reduces systemic toxicity and avoids substrate dependence while enabling in situ immune modulation. Studies in vivo show that macrophage-targeting piezoNPs combined with local US can effectively enhance immune activation and clear infections. This mitochondria-inspired, US-responsive nanoplatform offers a controllable, noninvasive strategy to boost innate immunity and holds promise for treating drug-resistant bacterial infections.

**Fig. 8.**
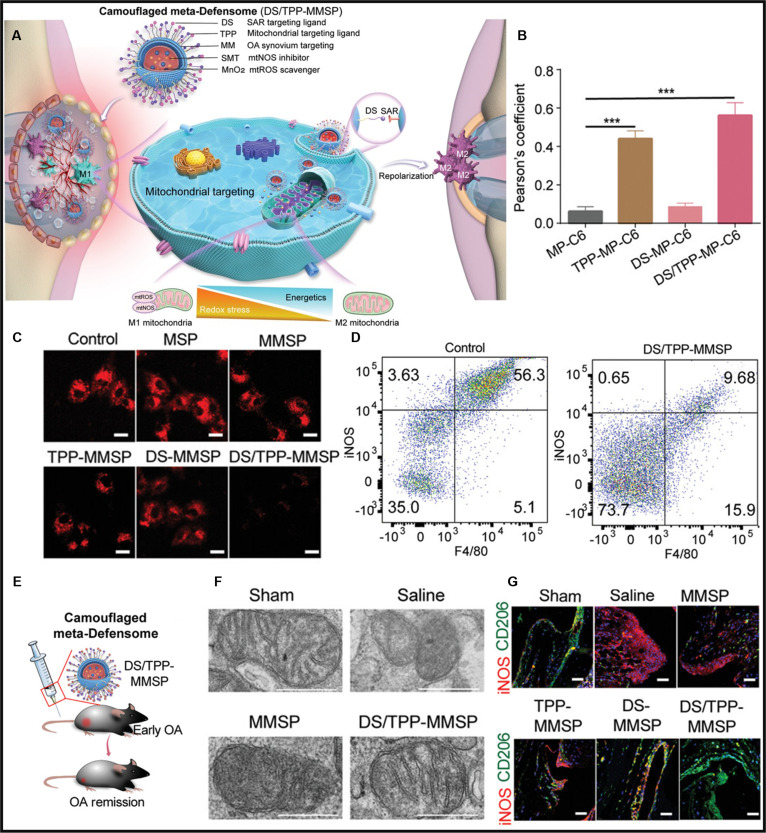
(A) Schematic of meta-Defensome composition and mitochondrial reprogramming in M1 synovial macrophages through mtROS scavenging and mtNOS inhibition. (B) Colocalization coefficient of C6-labeled multi-stage formulations with mitochondria in M1 macrophages. (C) mtROS levels of M1 macrophages decreased by meta-Defensomes treatment. (D) Ratio of M1 macrophages increased by meta-Defensomes treatment. (E) Schematics illustrating that meta-Defensomes treatment attenuates OA in model mice. (F) TEM images of the mitochondria of synovial macrophage from OA mice with different treatments. (G) Immunofluorescence of iNOS and CD206 in the synovium from OA mice with different treatments [184]. Copyright 2022, Wiley-VCH GmbH.

However, all the aforementioned ROS scavengers lack precise targeting of both macrophages and mitochondria, limiting their efficiency. Zhang’s group [184] developed a precisely targeted delivery system named meta-Defensome for ROS scavengers by metabolically engineering macrophage membranes and designing dextran sulfate (DS) for targeting and internalizing into the cytoplasm of M1 macrophages (Fig. 8A). Furthermore, the formulations were modified with tetraphenylphosphonium to enable mitochondrion-targeting capabilities (Fig. 8B). In this system, manganese dioxide nanoparticles (MnO_2_ NPs) were encapsulated to scavenge abnormal mtROS, while S-methylisothiourea (SMT), an iNOS inhibitor, was simultaneously incorporated to inhibit abnormal mitochondrial nitric oxide synthase (mtNOS) expression (Fig. 8C). This composite was shown to successfully reprogram the mitochondrial metabolism of M1 macrophages by scavenging mtROS and inhibiting mtNOS, thereby increasing TFAM expression and restoring aerobic respiration. As a result, the M1 macrophages were efficiently reprogrammed to the M2 phenotype in vitro, with high transformation efficiency (Fig. 8D). They further demonstrated that meta-Defensomes effectively suppressed OA progression in vivo via intravenous injection (Fig. 8E). After meta-Defensomes treatment, mitochondrial morphology improved with reduced swelling and more organized cristae, as well as increased area (Fig. 8F). Meanwhile, the recovery of mitochondrial dysfunction in synovial tissue successfully suppressed M1 polarization of synovial macrophages in mice, alleviating arthritis and cartilage destruction (Fig. 8G).

### Ion homeostasis: Balancing ionic gradients

Excessive mitochondrial Ca^2+^ accumulation, exacerbated by inflammatory signaling, induces persistent mitochondrial mPTP activation—a self-amplifying cycle where Ca^2+^ overload and mtROS reciprocally enhance each other, driving mitochondrial membrane depolarization and inflammatory escalation. To disrupt this pathological cross-talk, He et al. [185] engineered “nanogluttons” (PPTB NPs), mitochondrial-targeted nanoparticles combining a polymer-based dendrimer framework with PEG-TPP for organelle-specific delivery. These pH-responsive carriers encapsulate BAPTA-AM, a Ca^2+^ chelator that releases selectively in acidic inflammatory microenvironments. Upon macrophage internalization, PPTB NPs accumulate at mitochondria, where BAPTA-AM blocks pathological Ca^2+^ influx while preserving physiological Ca^2+^ oscillations. Through the dual modulation of mitochondrial bioenergetics and inflammatory cascades, PPTB NPs demonstrated therapeutic synergy by suppressing IL-1β/IL-6-driven inflammation while enhancing osteogenic repair in periodontitis models, exemplifying how precision mitochondrial engineering can resolve inflammation–metabolism interdependencies.

Lei et al. [186] designed the MSN–ethylenebis (oxyethylenenitrilo)tetraacetic acid (EGTA)/TPP-PEG nanoparticles termed METP NPs to regulate mitoCa^2+^ and phenotype in macrophages with the ability of METP NPs to target mitochondria with TPP and consume excessive calcium ions with EGTA, a calcium ion-binding group. They demonstrated that METP NPs effectively and selectively inhibited the abnormal increase of mitoCa^2+^ in macrophages of OA mice or LPS-induced cells in vitro, thus inhibiting the proinflammatory phenotype of bone marrow-derived macrophages by suppressing the proinflammatory-related metabolism. Those changes blocked the OA inflammatory cascades at an early stage and controlled the OA-induced structure destruction, thereby achieving a considerable therapeutic effect in alleviating OA in mice.

Although these pioneering studies demonstrated innovative strategies to mitigate macrophage inflammation driven by mitochondrial Ca^2+^ overload, their nanomaterial designs exhibited limited specificity in discriminating healthy versus dysfunctional mitochondria, as well as in achieving macrophage-selective targeting. Consequently, future efforts should focus on developing next-generation platforms capable of precise spatiotemporal control over mitochondrial Ca^2+^ homeostasis in macrophages, with enhanced cellular and organelle-level specificity.

### mtDNA surveillance: Intercepting inflammatory cascades

mtDNA has emerged as a central orchestrator of macrophage functional plasticity, bridging metabolic fidelity and inflammatory signaling. Recent advances have unveiled diverse strategies to intercept mtDNA-driven inflammatory circuits. By targeting distinct nodes of mtDNA generation, release, or recognition, these approaches recalibrate macrophage fate from pro-inflammatory to regulatory states, offering precision control over immune-metabolic diseases.

Among these strategies, multi-component nanomedicine platforms exemplify precision engineering to intercept mtDNA-driven inflammation at multiple levels, such as the multifunctional hydrogel system (CsA-TT-ENPP1@PEGA) engineered to coordinately target inflammation-driven dysregulation of mtDNA and its impact on cell function by addressing mtDNA leakage and cGAS-STING pathway activation [187]. This injectable platform incorporates cyclosporin A (CsA) to block mitochondrial mPTP, thereby preventing pathological mtDNA release into the cytosol. Concurrently, thiethylperazine (TT) enhances ABCC1-mediated cyclic GMP-AMP (cGAMP) efflux, while ENPP1 catalyzes cGAMP hydrolysis—synergistically resolving cytosolic nucleic acid accumulation. The localized delivery of CsA-TT-ENPP1@PEGA demonstrates a precise strategy to intervene in the mtDNA-cGAS-STING axis. While this approach was applied to stem cells, its ability to target different points of the mtDNA pathway highlights its potential for recalibrating macrophage polarization in the future.

Complementing such multi-component systems, natural product-based strategies offer alternative routes to decouple mtDNA release from inflammatory cascades. Baicalin is an active flavonoid isolated from *Scutellaria baicalensis* Georgi that has long been used as heat-clearing and detoxifying traditional Chinese medicinal herbs. Mechanistic studies reveal that baicalin maintains mitochondrial integrity under inflammatory stress, preventing the release of mtDNA into the cytosol [188]. This intervention prevents the formation of Z-DNA, a structure formed from oxidized mtDNA in the cytosol that activates ZBP1-dependent PANoptosis by triggering pyroptosis, apoptosis, and necroptosis. By blocking ZBP1–PANoptosome assembly, baicalin suppresses caspase activation, gasdermin pore formation, and mixed lineage kinase domain-like pseudokinase (MLKL) phosphorylation, thereby uncoupling mitochondrial damage from inflammatory cell death amplification. These actions collectively reprogram macrophages toward an immunoregulatory phenotype, attenuating cytokine storms and tissue injury. This work positions baicalin as a mitochondrial-targeted agent that recalibrates macrophage function through mtDNA-centric signaling, offering a therapeutic paradigm for inflammatory diseases driven by PANoptosis.

In contrast to this natural product-based strategy targeting mtDNA leakage, doxycycline exemplifies pharmacological repurposing of antibiotics for mitochondrial genomic regulation. Mechanistic studies demonstrate that doxycycline inhibits mitochondrial translation, a process essential for mtDNA replication and stability [189]. This disruption reduces both mtDNA abundance and its oxidized form (Ox-mtDNA), critical ligands for NLRP3 inflammasome assembly. Genetic silencing of mitochondrial methionyl-tRNA formyltransferase (Mtfmt), a key enzyme in mitochondrial translation, reproduced these effects. This confirmed that mtDNA depletion leads to reduced cytosolic Ox-mtDNA levels, which in turn diminishes caspase-1 activation and IL-1β secretion. The antibiotic’s ability to attenuate NLRP3-driven pathologies, such as LPS-induced systemic inflammation, underscores mtDNA synthesis as a pivotal checkpoint for macrophage inflammatory fate determination. This work repositions ribosomal-targeted antibiotics as precision tools to modulate mtDNA–immune crosstalk, offering novel therapeutic avenues for inflammatory diseases.

Expanding the toolkit, nucleic acid nanotechnologies offer gene-targeted control over mtDNA release mechanisms. Liu’s team [190] designed a siRNA-based mitochondrial intervention strategy to silence slc44a1, a critical transporter regulating choline uptake in macrophages. Delivered via a tetrahedral nucleic acid scaffold (“nano-windmill”), the siRNA down-regulates CTL1 expression, thereby reducing mitochondrial choline accumulation and subsequent mtDNA leakage. This dual-action mechanism suppresses NLRP3 inflammasome activation and pro-inflammatory cytokine release by blocking both mitochondrial stress and cytosolic DNA sensing. Collectively, mtDNA-centric strategies exemplify the therapeutic potential of mitochondrial–immune crosstalk modulation. Although current tools show promise, future strategies must enhance mitochondrial targeting in inflamed tissues and integrate mechanistic precision with clinical applicability through the use of stimuli-responsive nanomaterials, thereby enabling tailored regulation of macrophage fate via mtDNA modulation. Bridging mitochondrial biology with immunology and nanotechnology will unlock next-generation therapies to resolve inflammation at its metabolic source.

## Direct Strategies for Targeting of Mitochondria

Contemporary approaches to subcellular drug delivery predominantly involve bioengineering strategies. Therapeutic agents ranging from small molecules to nanocarriers are chemically or physically modified with organelle-directing groups [191]. Within this paradigm, direct mitochondrial targeting achieves molecular precision through 2 distinct approaches. The first involves covalent bonding of mitochondrial-homing ligands to drug molecules. The second utilizes intrinsically targeted compounds that naturally localize to mitochondria.

The operational superiority of direct strategies arises from their capitalizing on mitochondrial membrane biophysics. This strategy specifically relies on the synergistic exploitation of lipophilicity for bilayer penetration and cationic charge for Δψm-driven electrophoretic accumulation to overcome concentration gradients [192]. This physicochemical rationale underpins 4 principal targeting modalities: small-molecule vectors typified by delocalized lipophilic cations (DLCs), redox-active transition metal complexes with innate membrane permeability, bioactive peptides exhibiting mitochondrial membrane selectivity, and targeting material-based nanocarriers (Table 1). While organic small-molecule conjugates and metallodrugs constitute the 2 primary branches of direct targeting, their functional divergence lies in pharmacokinetic behavior. Small-molecule conjugates depend on attached targeting units, while metallodrugs incorporate targeting capability directly into their metal coordination sphere. Crucially, these direct modalities complement drug-mediated indirect strategies, particularly when dealing with chemically intractable therapeutics [193]. The choice between these strategies depends on molecular complexity. Direct targeting works best for chemically modifiable molecules, enabling efficient intervention. Indirect approaches better suit structurally challenging compounds through adaptable mechanisms.

**Table 1.** Targeting strategies: An inherent pillar of mitochondrial therapies

Classification	Targeting mechanism	Targeting site	Advantages	Disadvantages	Material	Refs.
Small molecules	Electrostatic interaction	MM	Mature use	Toxic; large cargo limited	TPP/Rhodamine/Dequalinium/Guanidine/Biguanide	[163,186,195,199]
Bioactive molecules	Electrostatic interaction; hydrophobic interaction	OMM\IMM	Tunability; ease of synthesis; high specificity; low toxicity	Large structure; low solubility; low permeability	SS/MPPs/MTS	[202–209]
Transition metal complexes	Electrostatic interaction; hydrophobic interaction	Adjustable	Photophysical properties; photothermal properties	Toxic; low efficiency	Ru/Mn/Pt/Cu/Ni/Ir/Zn	[195,210–213]
Nanocarriers	Adjustable	Adjustable	Low toxicity; rich functions; higher efficiency	High cost; difficult to synthesize	CQD/BS-NPs	[184,215–217]

### Small molecules

The mitochondrial transmembrane potential constitutes a cornerstone for organelle-targeting strategies, as even subtle perturbations in this electrochemical gradient profoundly impact mitochondrial homeostasis. DLCs are a class of low-molecular weight compounds and accumulate in mitochondria by leveraging on the electrochemical gradient that exists across the cytoplasm and the IMM [194]. Most of the existing mitochondria-targeting small molecules including TPP, rhodamine, dequalinium, and guanidine/biguanide are DLCs [195]. The lipid solubility of these molecules enables them to cross the cell membrane and mitochondrial membrane, and the positive charge enables them to enter mitochondrial matrix (MM) under the action of the mitochondrial membrane potential, endowing them with mitochondrial targeting ability [196]. The use of small molecules for mitochondrial targeting is relatively well established due to their long history of use in various biological applications. However, the use of DLCs may be limited by their toxicity at high concentrations as their accumulation in mitochondria may disrupt mitochondrial membrane potential, leading to cell toxicity [197]. While being good carriers for lipophilic or small polar molecules, the effectiveness of each DLC at delivering larger cargoes may still be dependent on the overall size and charge of the conjugates [198].

TPP derivatives dominate current mitochondrial targeting approaches due to their unique pharmacochemical profile. Through covalent conjugation with bioactive molecules or functionalization of nanocarriers, these amphiphilic compounds achieve precise subcellular localization. Lei et al. [186] demonstrated EGTA-loaded MSN prepared using TPP-modified PEG effectively targeting mitochondria in macrophages. The advantages of TPP-based mitochondrial targeting over other approaches for mitochondrial delivery of small molecules include the stability of the TPP moiety in biological systems, a combination of lipophilic and hydrophilic property, the relatively simple synthesis and purification, the low chemical reactivity toward cellular components, and their lack of light absorption or fluorescence in the visible or near-infrared (NIR) spectral region [199]. Emerging evidence further reveals the functional duality of mitochondrial-targeted small molecules beyond mere localization. Pioneering work by Wang et al. [163] identified IR-61, an anti-inflammatory cyanine derivative that selectively accumulates in adipose tissue macrophages. This mitochondria-targeted compound exhibits dual therapeutic efficacy, simultaneously inhibiting proinflammatory activation and improving metabolic dysfunction. These effects illustrate how precision-designed small molecules can function as both organelle-specific regulators and disease-modifying therapeutics.

### Bioactive molecules

Peptides have emerged as versatile biological tools owing to their molecular precision, high target affinity, and synthetic accessibility through advanced solid-phase methodologies [200]. In mitochondrial pharmacology, engineered peptides offer distinct advantages over conventional lipophilic cations, with 3 principal classes demonstrating organelle-specific tropism: Szeto–Schiller (SS) peptides, mitochondrial penetrating peptides (MPPs), and mitochondrial targeting sequence (MTS) peptides—each exploiting unique mechanisms for subcellular navigation.

SS peptides have the ability to permeate the plasma membrane, selectively accumulate in the IMM of mitochondria, and scavenge ROS [201]. SS peptides can freely penetrate cells at physiological pH as their cellular uptake is energy-independent, but the exact mechanism of targeting mitochondria remains unclear [202]. It has been postulated that SS peptides concentrate at greater levels in the IMM compared to the matrix due to the electrostatic attraction between the cationic peptide with the phosphate head groups on cardiolipin in the IMM [198]. Recent studies demonstrate that SS-31 in macrophages concurrently suppresses mtROS generation and down-regulates scavenger receptors CD36/LOX-1, thereby preventing oxidized low-density lipoprotein-induced foam cell formation [203]. This dual-action mechanism highlights SS-31’s therapeutic potential in mitigating both inflammatory cascades and cholesterol accumulation, positioning it as a promising multifaceted intervention strategy.

MPPs are a type of widely utilized mitochondria-targeting molecules that have been discovered in 2008 [204]. MPPs have excellent cell permeability and mitochondrial targeting because they have alternating cationic and hydrophobic residues [204]. At the same time, Horton et al. [205] consider that the uptake of MPPs by cells seems to be independent of the endocytosis pathway, bypassing endosomal or lysosomal sequestration, which also increases the chance of MPPs reaching mitochondria. Their modular design allows systematic optimization of pharmacokinetic profiles while maintaining synthetic simplicity, which is particularly valuable for in vivo applications [206]. Building on this foundation, a recent mitochondria-targeting and membrane-penetrating peptide amphiphile (MMPA)-based nanoplatform was engineered as a multifunctional derivative [207]. This system leverages MMPA’s charge-mediated siRNA complexation to achieve mitochondrial delivery of therapeutic siRNA, specifically down-regulating mtDNA-encoded proteins like ATP6. Crucially, the induced mitochondrial damage activates innate immunity, leading to the repolarization of TAMs into the antitumor M1 phenotype.

The third paradigm, MTS peptides, mirrors endogenous mitochondrial protein import machinery. These N-terminal sequences form amphipathic α-helices where hydrophobic faces and cationic surfaces synergistically engage translocation complexes [206]. While their structural bulk and membrane impermeability pose delivery challenges, recent nanotechnological innovations circumvent these limitations [194]. In this regard, the mitochondria-regulated information processing nanosystem developed by Che et al. [208] incorporates a cysteine-containing peptide that directs the nanosystem into the MM. Remarkably, this nanosystem targets αvβ3-overexpressing hepatic stellate cells (HSCs), where it selectively binds to the integrin receptor αvβ3 and is internalized by HSCs. This strategy overcomes the permeability limitations of MTS across the cell membrane, achieving dual-targeting at both the cellular and organellar levels. Parallel advancements by Zuo et al. [209] integrated MTS with liposomes, leveraging the enhanced endocytic capacity of macrophages in the pro-inflammatory phenotype to achieve a similar dual-targeting effect at both the cellular and organellar levels.

Collectively, these peptide-based strategies underscore a critical design paradigm: Integrating bioinspired molecular mimicry with programmable synthetic modifications transcends conventional drug delivery limitations, permitting spatially precise interventions at the organelle level.

### Transition metal complexes

Transition metal complexes demonstrate intrinsic mitochondrial tropism through synergistic physicochemical attributes—elevated cationic charge density facilitates Δψm-driven electrophoretic accumulation, while optimized lipophilicity enables bilayer penetration [210]. Research has revealed that various transition metal complexes, such as those containing Ru, Mn, Pt, Cu, Ni, Ir, and Zn, exhibit the capability to specifically target mitochondria [195]. Notably, their tunable photophysical profiles render them superior photosensitizers for spatiotemporally controlled photodynamic interventions, particularly in oncological applications where mitochondrial disruption synergizes with ROS generation [211]. Nevertheless, the therapeutic promise of these transition metal complexes is counterbalanced by inherent pharmacological challenges. Cationic amphiphilicity, while crucial for mitochondrial targeting, predisposes complexes to nonspecific interactions with anionic membrane components of other organelles—a phenomenon exacerbated in pathological microenvironments with altered membrane potentials. Furthermore, excessive intramitochondrial accumulation disrupts proton gradient homeostasis, triggering apoptotic cascades that manifest as concentration-dependent cytotoxicity [212]. Current research frontiers focus on engineering charge-delocalized architectures and stimuli-responsive ligands to decouple therapeutic efficacy from off-target effects, aiming to transform these inherent liabilities into precision-controlled therapeutic modalities. In an exemplar engineering approach, the copper-depleting moiety (CDM) within the CDM@MUiO-DP@MCHM platform capitalizes on transition metal redox properties while mitigating off-target risks [213]. This copper-targeting agent leverages mitochondrial tropism to induce localized copper depletion, triggering energy collapse via membrane potential dissipation and immunomodulation through tumor microenvironment remodeling. Macrophage-derived hybrid membrane encapsulation restricts CDM’s activity to malignant mitochondria, reducing protumoral macrophage functions through tumor–stromal interactions.

### Nanocarriers

Contemporary nanocarrier designs for mitochondrial delivery primarily employ bioorthogonal conjugation strategies, integrating cationic homing ligands or amphipathic peptides to achieve subcellular precision. This modular engineering approach synergistically enhances theranostic efficacy while mitigating ligand-induced cytotoxicity—particularly evident in redox-sensitive systems where controlled payload release coincides with mitochondrial activation [214]. Advanced platforms further incorporate microenvironment-responsive elements, enabling hierarchical targeting through pH- or enzyme-triggered charge reversal mechanisms that sequentially address cellular, subcellular, and organellar barriers [215]. The strategic coupling of functional nanomaterials with mitochondrial therapeutics creates multifunctional synergies. TPP-functionalized biodegradable silica nanoparticles (BS-NPs) exemplify this approach, demonstrating enhanced protein packaging efficiency, enzymatic protection, and spatiotemporal release kinetics compared to conventional vector systems [216]. Nevertheless, such sophistication escalates synthetic complexity, demanding meticulous optimization of ligand density, surface charge distribution, and biodegradation profiles to balance functionality with clinical translatability. Emerging paradigms challenge the necessity of exogenous targeting moieties. Pioneering work by Li et al. [217] revealed that certain carbon quantum dots (CQDs) synthesized via single-step hydrothermal protocols exhibit intrinsic organelle tropism. These zero-dimensional nanomaterials achieve dual-mode mitochondrial localization and real-time bioimaging without supplementary ligands, their inherent photophysical properties and surface charge distribution serendipitously mimicking endogenous trafficking patterns [218]. Nanocarriers offer a distinct advantage over the previous 3 approaches by enabling multiplexed targeting, allowing for hierarchical targeting of both macrophages and mitochondria [184]. This enables precise, cascade-like therapeutic interventions, enhancing the accuracy and efficacy of treatment strategies.

## Conclusion and Outlook

**Fig. 9.**
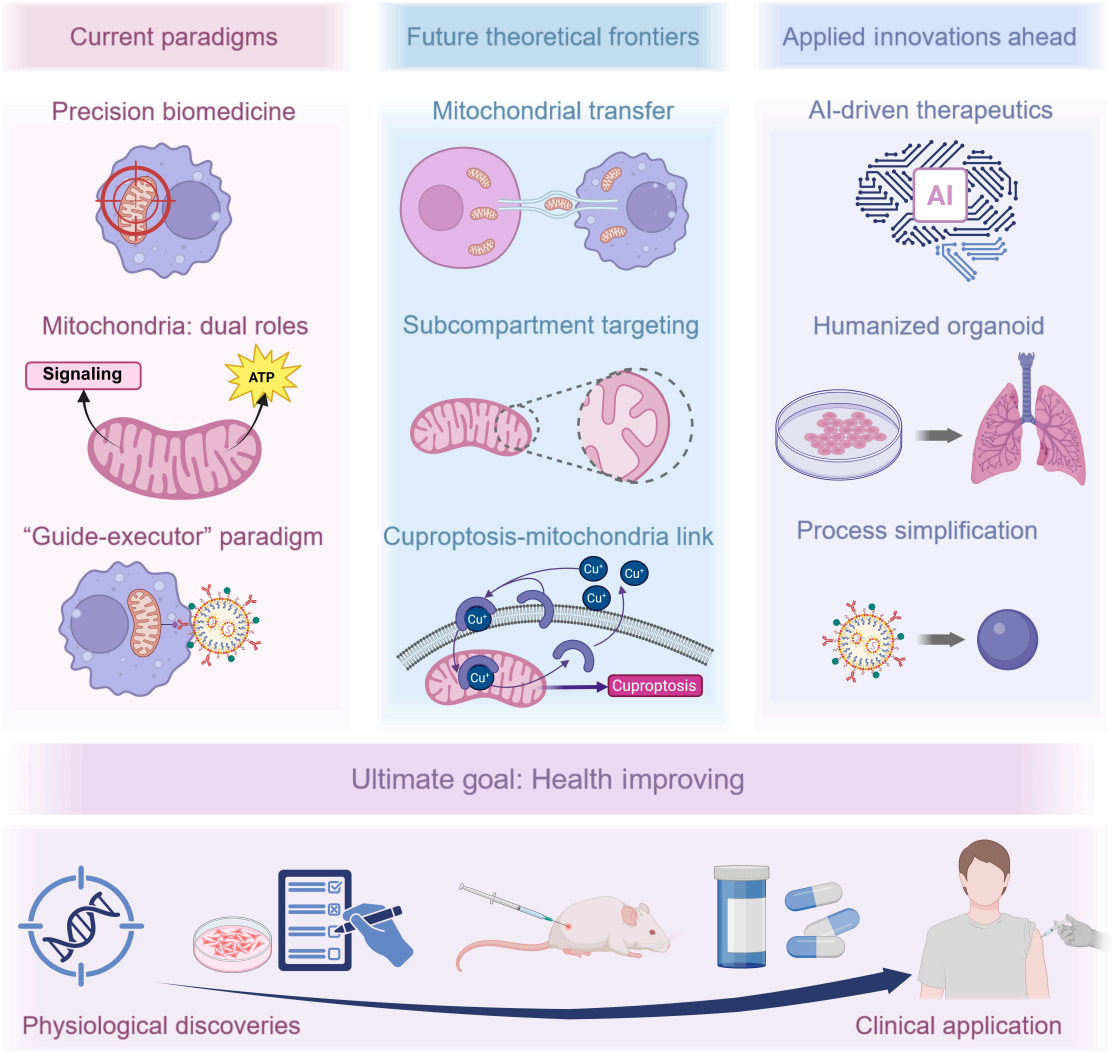
Perspectives on mitochondrial targeting strategies for macrophage fate modulation. Current “guide-executor” paradigms, leveraging mitochondria’s dual functionality, have advanced precision biomedicine. Looking to the future, the integration of theoretical depth and technological innovation will propel mitochondrial targeting strategies to the ultimate goal of health improving (created with BioRender.com).

The emergence of precision biomedicine has revolutionized cellular engineering through unprecedented control over cellular fate determination. Within this paradigm, mitochondria have gained renewed research attention following the discovery of their dual functionality as both energy generators and signaling integrators, particularly in the context of macrophage fate dysregulation (Fig. 9). Here, we present a systematic classification of mitochondrial intervention strategies, distinguishing indirect systemic approaches from direct mitochondrial targeting methods. To establish therapeutic rationale, we first delineate the multifaceted components of mitochondrial homeostasis, emphasizing their differential contributions to physiological maintenance versus pathological progression in macrophage populations. Current therapeutic development employs a posttargeting modulation framework that combines organelle-specific delivery systems with mitochondrial-normalizing agents. The most advanced form of this “guide-executor” paradigm occurs in the CRISPR-Cas9 system, which utilizes single-guide RNA (sgRNA) to recognize target DNA sequences for precise localization, and then performs the cutting function by Cas9 nuclease [219]. While existing mitochondrial targeting strategies implement a primitive version of this dual-component system, our analysis reveals opportunities for developing next-generation platforms that achieve spatiotemporal precision comparable to contemporary genome-editing technologies.

The evolving theoretical framework of mitochondrial homeostasis has established a conceptual foundation that not only informs targeted therapeutic development but also directs strategic research priorities. Central to this advancement is the “guide-executor” regulatory paradigm, which operationalizes the translational potential of mitochondrial homeostasis research through mitochondria-mediated epigenetic reprogramming of macrophage fate. This paradigm shift, emerging from the recognition of mitochondria as information processing hubs, demonstrates how fundamental discoveries in organelle biology can drive therapeutic innovation, exemplified by mitochondria-targeted interventions in inflammatory disorders. Converging evidence identifies 4 actionable frontiers in mitochondrial regulation: (a) The discovery of intercellular mitochondrial transfer mechanisms has necessitated reevaluation of conventional cell-autonomous perspectives on mitochondrial regulation [220]. This intercellular trafficking system positions mitochondria as critical mediators of cellular crosstalk, thereby enabling novel therapeutic approaches that transcend single-cell targeting limitations [221]. Donor cell-derived mitochondrial modulation strategies now complement classical “guide-executor” interventions through their capacity to reprogram recipient macrophage populations via horizontal organelle transfer. (b) The structural and functional compartmentalization of mitochondria (inner membrane, cristae, matrix) presents both challenges and opportunities for therapeutic targeting [222]. Distinct biochemical microdomains within these subcompartments maintain specialized regulatory networks that govern mitochondrial metabolism and signaling. This architectural complexity underscores the imperative to develop spatially resolved intervention strategies through advanced imaging techniques and nanoscale biosensors, particularly for precision modulation of cristae remodeling processes that dictate cellular survival decisions. (c) Emerging mitochondrial death pathways, particularly cuproptosis mediated by copper ion flux, are expanding the therapeutic landscape beyond conventional M1/M2 polarization paradigms [223]. The mitochondrial copper transporter SLC31A1 has emerged as a critical regulator of this process through its modulation of lipoylated TCA cycle enzymes, revealing novel targets for macrophage fate manipulation in copper-associated pathologies [120]. Future research must deepen insights into mitochondrial regulatory mechanisms, such as how molecular pathways in mitochondria control macrophage behavior, and broaden exploration of uncharted mitochondrial processes, including interactions with understudied ions or organelles. (d) The advancement of AI has introduced powerful tools for analyzing mitochondrial dysfunction and its connection to macrophage behavior and disease. AI can accurately identify distinct mitochondrial abnormalities such as oxidative stress, disrupted dynamics, and metabolic imbalance. It can also help reveal how these specific conditions shape macrophage polarization and function during disease progression [224]. This capability could greatly accelerate the discovery of precise mitochondrial-targeted interventions.

The translation of mitochondrial biology into clinically actionable strategies demands the development of innovative therapeutic platforms that synergize precision targeting, biocompatibility, and scalable manufacturing. This paradigm shift requires transitioning from conventional empirical approaches to rational design frameworks. This requires maximizing therapeutic efficacy while minimizing off-target effects, adhering to the clinically pivotal “precision-over-redundancy” principle. Three pivotal directions are poised to reshape mitochondrial therapeutics: (a) AI has emerged as a transformative enabler in this domain. For instance, graph neural networks trained on mitochondrial proteomic datasets can predict nanocarrier–mitochondrial membrane interactions with atomic-level precision, enabling de novo design of organelle-specific delivery systems [225]. Similarly, AI-driven analysis of mitochondrial metabolic networks identifies drug combinations that simultaneously widen therapeutic windows and limit off-target impacts. This represents a key advance for managing inflammatory diseases. (b) A persistent translational challenge arises from interspecies disparities in preclinical models, particularly in mitochondrial dynamics and drug metabolism. To overcome this limitation, humanized organoid platforms are emerging as a transformative tool to replicate human-specific mitochondrial–immune crosstalk [226]. These systems integrate patient-derived cells with engineered microenvironments, enabling high-fidelity prediction of human responses through multi-scale analysis functional outcomes. (c) Simplifying synthesis processes for mitochondrial-targeted agents is equally critical. Advances in small-molecule drug design integrates computer simulation and machine learning. This approach creates stable, drug-like compounds that emulate peptide targeting, significantly improving clinical translation potential [227].

The advancement of biomedicine fundamentally depends on the reciprocal reinforcement of theoretical discovery and translational innovation, a paradigm exemplified by recent breakthroughs in mitochondrial immunometabolism. Mitochondrial-targeted macrophage regulation exemplifies this synergy through 3 evidence-based translational pathways: (a) identifying macrophage-specific mitochondrial biology questions through expanded theoretical research, (b) addressing these challenges by integrating innovative technologies with strategic design, and (c) bridging molecular and physiological discoveries in mitochondrial biology with human health. By aligning theoretical depth with technological innovation, mitochondrial-targeted therapies hold promise for revolutionizing precision medicine and restoring immune homeostasis in disease.
